# Fanconi anemia DNA crosslink repair factors protect against LINE-1 retrotransposition during mouse development

**DOI:** 10.1038/s41594-023-01067-8

**Published:** 2023-08-14

**Authors:** Nazareno Bona, Gerry P. Crossan

**Affiliations:** MRC Laboratory of Molecular Biology, Cambridge, UK

**Keywords:** Biochemistry, Developmental biology, Chromosomes, Genomic instability, Tumour-suppressor proteins

## Abstract

Long interspersed nuclear element 1 (LINE-1) is the only autonomous retrotransposon in humans and new integrations are a major source of genetic variation between individuals. These events can also lead to de novo germline mutations, giving rise to heritable genetic diseases. Recently, a role for DNA repair in regulating these events has been identified. Here we find that Fanconi anemia (FA) DNA crosslink repair factors act in a common pathway to prevent retrotransposition. We purify recombinant SLX4-XPF-ERCC1, the crosslink repair incision complex, and find that it cleaves putative nucleic acid intermediates of retrotransposition. Mice deficient in upstream crosslink repair signaling (FANCA), a downstream component (FANCD2) or the nuclease XPF-ERCC1 show increased LINE-1 retrotransposition in vivo. Organisms limit retrotransposition through transcriptional silencing but this protection is attenuated during early development leaving the zygote vulnerable. We find that during this window of vulnerability, DNA crosslink repair acts as a failsafe to prevent retrotransposition. Together, our results indicate that the FA DNA crosslink repair pathway acts together to protect against mutation by restricting LINE-1 retrotransposition.

## Abstract (web summary)

Here the authors show that Fanconi anemia repair proteins protect against LINE-1 retrotransposition, particularly during early development when the primary protective pathway of transcriptional silencing is inactive.

## Main

Long interspersed nuclear element 1 (LINE-1) elements are autonomous transposons in humans and make up 17% of the genome^1^. LINE-1 retrotransposition occurs by a ‘copy and paste’ mechanism; the RNA intermediate is reverse-transcribed and integrates at a new genomic location^2–5^. LINE-1 elements have shaped the evolution of the human genome but are frequently deleterious causing insertional mutagenesis, transcriptional dysregulation and genome instability^6,7^. New insertions are frequently pathogenic causing inherited diseases or contributing to somatic diseases^7–11^.

Organisms restrict LINE-1 retrotransposition by limiting its transcription and translation^12,13^. DNA methylation and chromatin modification play critical roles in restricting LINE-1 (refs. ^12–15^). Germline-specific restriction factors, for example the Piwi-interacting RNA (piRNA) system, are critical to restrict LINE-1 and maintain fertility^12,14,16^. However, recent evidence has implicated DNA repair in promoting and suppressing LINE-1 integration. This suggests that if a LINE-1 element evades transcriptional silencing, DNA repair can limit integration. Factors involved in a wide range of repair processes, but particularly in replication fork stability, have been implicated^17–23^. Of interest is the Fanconi anemia (FA) pathway, which repairs DNA interstrand crosslinks (ICLs), as multiple factors from this pathway suppress retrotransposition; however, it is unclear how FA proteins act to suppress LINE-1 retrotransposition^13,20,21,24^.

While these studies clearly demonstrate that DNA repair can restrict LINE-1 elements in tissue culture, the physiological importance of these restraint mechanisms is unknown. Despite effective retrotransposon transcriptional silencing, new insertions occur both in somatic cells and in the germline; therefore, DNA repair could act to limit retrotransposition in vivo^16^. Physiological processes required for development render cells more susceptible to LINE-1 integration^9,16,24^. Perhaps the best examples of this are early zygote and germ cell development in which genome-wide epigenetic reprogramming occurs and LINE-1 transcriptional silencing is attenuated^25–27^. Therefore, DNA repair may play a particularly important role in these situations.

We use reverse genetics to show that DNA crosslink repair factors act in a common pathway to restrict LINE-1 retrotransposition. Moreover, we find that a reconstituted recombinant FA repair incision complex can cleave putative intermediates of retrotransposition. We show that all stages of DNA crosslink repair, defective in the human disease FA, are required to prevent retrotransposition in mice. DNA crosslink repair-deficient mice accumulate LINE-1 integrations in an array of somatic tissues with male germ cells having the highest levels. Finally, we find that early zygotic development is particularly dependent on DNA crosslink repair to prevent LINE-1 retrotransposition.

## Results

### DNA repair promotes or restrains LINE-1 retrotransposition

Previous studies have identified DNA repair factors that either promote or suppress LINE-1 retrotransposition^13,19,28,29^. It is proposed that DNA double-strand break (DSB) repair and replication fork stability factors play critical roles in suppressing retrotransposition^21^.

**Fig. 1:**
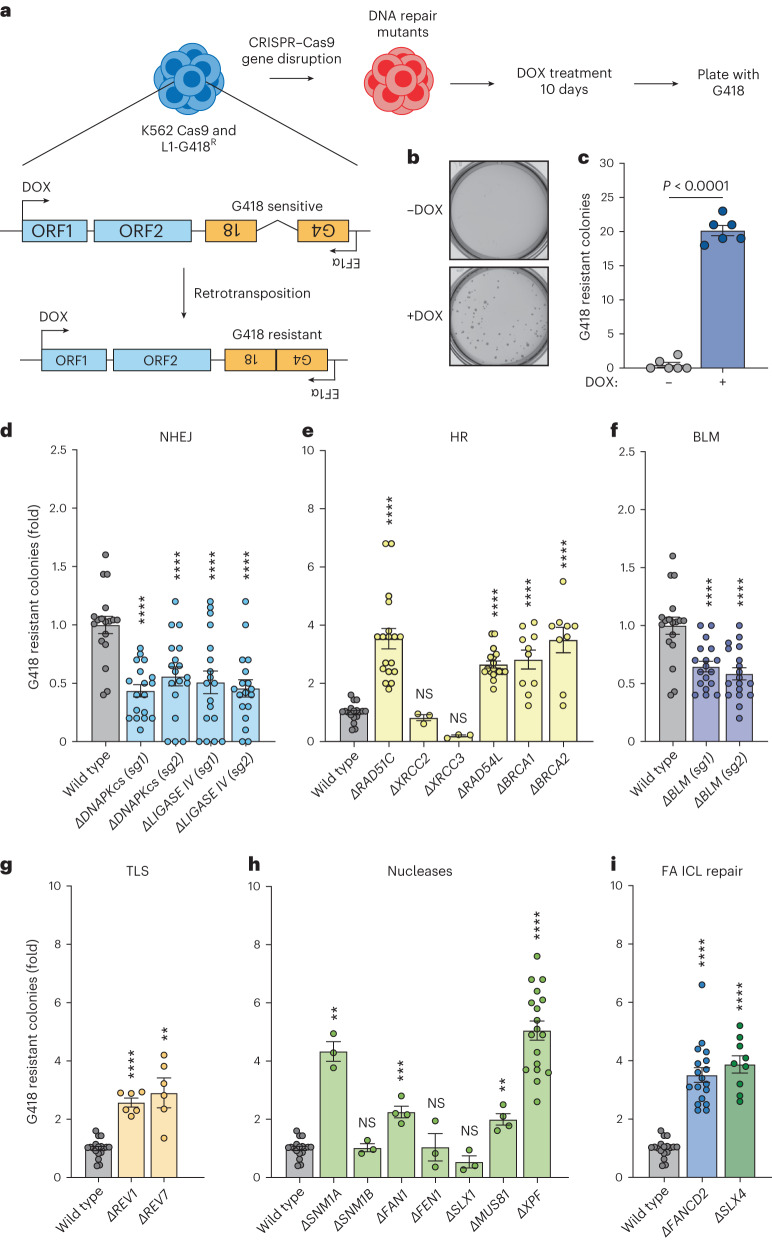
DNA repair factors regulate LINE-1 retrotransposition. **a**, Schema of the LINE-1 reporter (L1-G418^R^) used in K562 cells and the strategy used to quantify retrotransposition events. Cells carry a LINE-1 reporter with a G418 resistance cassette in the opposite orientation to the LINE-1 element. The G418 cassette is interrupted with an intron in the orientation of the LINE-1 element. Therefore, the LINE-1 transcript will undergo splicing and, following integration, the noninterrupted G418 cassette will be expressed. **b**, Image of K562 L1-G418^R^ colonies grown in semisold methylcellulose media following treatment with DOX and selection with G418. **c**, Quantification of K562 L1-G418^R^ G418-resistant colonies following DOX treatment. *n* = 6 independent experiments. **d**–**i**, As in **c** but for indicated mutants. Each dot corresponds to an independent experiment. **d**, NHEJ mutants: *ΔDNA-PKcs* (sg1), *ΔDNA-PKcs* (sg2), *ΔLIGASEIV* (sg1), *ΔLIGASEIV* (sg2). **e**, Homologous recombination (HR) mutants: *ΔRAD51C*, *ΔXRCC2*, *ΔXRCC3*, *ΔRAD54L*, *ΔBRCA1*, *ΔBRCA2*. **f**, *BLM*: *ΔBLM* (sg1), *ΔBLM* (sg2). **g**, Translesion synthesis (TLS): *ΔREV1*, *ΔREV7*. **h**, Nucleases: *ΔSNM1A*, *ΔSNM1B*, *ΔFAN1*, *ΔFEN1*, *ΔSLX1*, *ΔMUS81*, *ΔXPF*. **i**, FA DNA interstrand crosslink repair (FA ICL repair): *ΔFANCD2*, *ΔSLX4*. DNA repair mutant K562 cells were generated by CRISPR–Cas9 gene disruption. Sg1 and sg2 represent sgRNAs targeting different exons of the gene of interest. Each dot represents an independent experiment. Data represent mean and s.e.m. Unless otherwise specified, *P* values were calculated by a two-tailed Mann–Whitney *U*-test (NS, no significant *P* > 0.05, *P ≤ 0.05, **P ≤ 0.01, ***P ≤ 0.001, ****P ≤ 0.0001). Source data

We confirm these previous results but also ask if other classes of DNA repair factors regulate LINE-1 retrotransposition. We used a previously reported LINE-1 retrotransposition assay in the K562 cell line used to perform LINE-1 genome-wide screens^4,13^. It is an advantage that all mutants were generated from one parental line, allowing comparison between different mutants. We used genetic knockouts rather than small-interfering RNA allowing quantitative comparisons between mutants but also the generation of double mutants to perform classical genetic studies. A doxycycline (DOX)-inducible promoter drives the expression of the LINE-1 cassette; hence, cells will only be G418 resistant after retrotransposition (Fig. 1a). We showed that LINE-1 retrotransposition was dependent on DOX treatment (Fig. 1b,c). We then used CRISPR–Cas9 to generate cell lines deficient in factors required for DNA DSB repair (Supplementary Fig. 1–4). We first disrupted DNA-PKcs and LIGASE IV, components of nonhomologous end joining (NHEJ) and found that the LINE-1 integration frequency was reduced in agreement with previous reports (Fig. 1d and Supplementary Fig. 1a–f)^30–32^.

We disrupted *RAD51C*, *XRCC2* and *XRCC3*, three *RAD51* paralogues critical for homologous recombination (Supplementary Fig. 2a,b,e,g–j). LINE-1 integration frequency was increased in the absence of RAD51C but was unaffected by the loss of either XRCC2 or XRCC3 (Fig. 1e). Similar to RAD51C, the loss of BRCA1, BRCA2 or RAD54L led to an increase in the frequency of integration events (Fig. 1e and Supplementary Fig. 2c,d,f). It has been proposed that BRCA1 restricts LINE-1 retrotransposition by protecting the stability of the replication fork^17,21^. We have expanded on these findings to show that this requirement is not generalizable to all homologous recombination components. Moreover, we tested whether Bloom syndrome helicase (BLM), which can resolve the Holliday junction intermediate of homologous recombination, prevented retrotransposition. We found that the loss of BLM led to a decrease in LINE-1 integration frequency consistent with the hyper-recombinogenic nature of *BLM*-deficient cells (Fig. 1f and Supplementary Fig. 1g–i)^33^.

We tested whether other replication fork stability factors suppress retrotransposition. A critical mechanism used by cells to prevent replication fork collapse when impediments to replication are encountered is known as DNA damage tolerance^34^. Hence, we generated K562 cells deficient in REV1 and REV7 (Supplementary Fig. 3a–c). Loss of either of these factors led to increased LINE-1 integration frequency (Fig. 1g), suggesting there is a general requirement for factors involved in replication fork protection to prevent LINE-1 retrotransposition.

**Extended Data Fig. 1:**
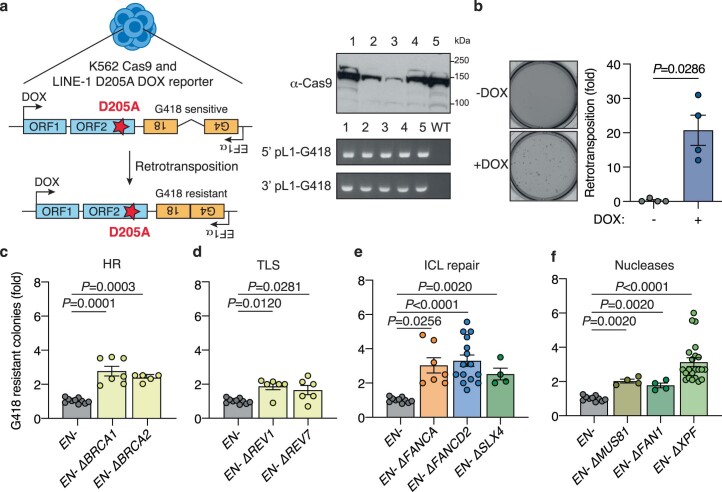
Fanconi anemia DNA crosslink repair factors restrict endonuclease-independent integration events. **a)** Schema illustrating the L1(D205A)-G418^R^ retrotransposition assay in K562 cells. ORF2 endonuclease activity was disrupted by a point mutation (D205A) generated by site-directed mutagenesis. Top, immunoblot for Cas9 in K562 cells. Bottom, PCR validating integration of the L1(D205A)-G418^R^ reporter (both ends of the construct) in genomic DNA extracted from the K562 cells expressing Cas9 (representative data from 2 independent experiments). **b)** K562 cells carrying the endonuclease dead L1(D205A)-G418^R^ (EN-) reporter were treated with DOX and then plated into selective methylcellulose media (containing 1 mg/mL G418). Colonies were quantified after 10 days. Each dot corresponds to an independent experiment. **c-f)** DNA repair-deficient mutants on the L1(D205A)-G418^R^ (EN-) background were assayed. Each dot corresponds to an independent experiment: **c)** Homologous recombination (HR), **d)** Translesion synthesis (TLS), **e)** Interstrand crosslink (ICL) repair and **f)** Nucleases. Data represent mean and s.e.m. Unless otherwise specified, *P* values were calculated by two-tailed Mann–Whitney U-test. Source data

The endonuclease activity of LINE-1 ORF2p is required for efficient retrotransposition; however, when this activity is lost, retrotransposition occurs with much lower efficiency^31,32^. We generated DNA repair-deficient K562 cell lines in an ORF2p endonuclease-deficient background (Extended Data Fig. 1 and Supplementary Fig. 5). We found that BRCA1 and BRCA2 suppress ORF2p endonuclease-independent integrations as previously reported (Extended Data Fig. 1c)^21^. REV1 and REV7 were both required to prevent such events (Extended Data Fig. 1d). This agrees with the proposal that LINE-1 could use either the free 3′ OH group of an Okazaki fragment to initiate reverse transcription, or breaks that occur when replication forks collapse^17^.

As nucleases convert stalled replication forks into breaks, we asked what effect their loss would have on LINE-1 integration. We generated cell lines lacking nucleases required for the maintenance of genome stability (Supplementary Fig. 3d–n). Four nucleases limited nuclease proficient LINE-1 retrotransposition: SNM1A, FAN1, MUS81 and XPF (Fig. 1h). We generated the same repair mutants in the ORF2p endonuclease-dead background (Supplementary Figs. 3l and 5g–j). To our surprise, rather than being required for these events FAN1, MUS81 and XPF limited ORF2p endonuclease-independent integrations (Extended Data Fig. 1f). This reveals that a subset of DNA repair-associated endonucleases is involved in LINE-1 nucleic acid metabolism. Rather than driving retrotransposition in the absence of LINE-1 endonuclease activity or converting stalled replication intermediates into substrates for retrotransposition, these nucleases actually reduce the frequency of LINE-1 integration events.

*XPF*-deficient cells exhibited an increase in the LINE-1 integration frequency (Fig. 1h). XPF is the nuclease component of the XPF-ERCC1 heterodimeric structure-specific endonuclease critical for multiple DNA repair transactions^35^. Biochemically, XPF-ERCC1 exhibits nuclease activity on splayed arms, 3′ overhangs and replication fork-like structures^36^. It was therefore surprising that other nucleases with similar biochemical activities (for example, SLX1) did not limit retrotransposition (Fig. 1h).

This led us to consider how XPF-ERCC1 was channeled into the suppression of LINE-1. XPF functions in multiple repair pathways; however, we found that FANCD2, SLX4 and RAD51C suppressed LINE-1 integration. It was previously hypothesized that FANCD2 protects the replication fork by limiting access to 3′ OH groups on the lagging strand thereby suppressing LINE-1 integration^17^. Additionally, SLX4 was shown to prevent the accumulation of LINE-1 intermediates in the cytoplasm and induction of cGAS-STING^20^. As these four factors act in FA DNA ICL repair, it is plausible that this common function is important to limit LINE-1 retrotransposition.

### Interstrand crosslink repair suppresses retrotransposition

**Fig. 2:**
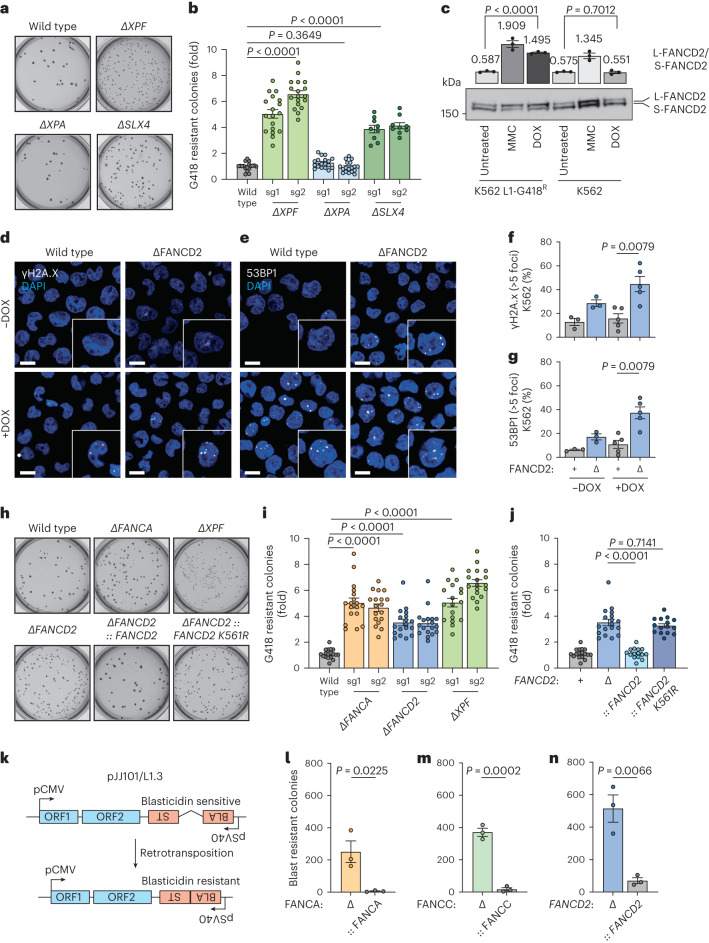
Interstrand crosslink repair factors restrict LINE-1 retrotransposition and preserve genomic stability. **a**, Image of K562 L1-G418^R^ wild-type and mutant colonies grown in semisold methylcellulose media following DOX treatment and G418 selection. **b**, L1 retrotransposition measurement using K562 L1-G418^R^ wild-type and mutant cells (sg1 and sg2, two different sgRNAs). Each dot corresponds to an independent experiment. **c**, Representative western blot of FANCD2 following 48 h expression of L1-G418^R^ in K562 cells and 24 h MMC treatment, *n* = 3 independent experiments, *P* values were calculated by a two-tailed unpaired *t*-test. **d**, Representative images of γ-H2A.x foci in K562 L1-G418^R^ cells before and after 48 h DOX treatment. Scale bars, 10 μm. **e**, Representative images of 53BP1 foci in K562 L1-G418^R^ cells before and after 48 h DOX treatment. Scale bar, 10 μm. **f**, Percentage of K562 L1-G418^R^ cells with more than five foci. Each dot corresponds to an independent experiment. **g**, Percentage of K562 L1-G418^R^ cells with more than five foci. Each dot corresponds to an independent experiment. **h**, Image of K562 L1-G418^R^ wild-type and mutant colonies grown in semisold methylcellulose media following DOX treatment and G418 selection. **i**, L1 retrotransposition measurement using K562 L1-G418^R^ wild-type and mutant cells (sg1 and sg2, two different sgRNAs), *n* = 18 independent experiments. **j**, L1 retrotransposition measurement using L1-G418^R^ in K562 cells (wild-type, *FANCD2* mutant and FANCD2 complemented cell lines). Each dot represents data from one independent experiment. **k**, Schema of the pJJ101/L1.3 reporter used in human fibroblasts. **l**–**n**, L1 retrotransposition measurement using pJJ101/L1.3 reporter in: PD220 (*FANCA*^*−/−*^) and complemented line (**l**), PD331 (*FANCC*^−/−^) and complemented line (**m**) and PD20 (*FANCD2*^−/−^) and complemented line (**n**), *n* = 3 independent experiments, *P* values were calculated by a two-tailed unpaired *t*-test. Data represent mean and s.e.m. Unless otherwise specified, *P* values were calculated by a two-tailed Mann–Whitney *U*-test. Source data

XPF-ERCC1 is critical for both nucleotide excision repair (NER) and FA repair^37–39^. We formally tested which role of XPF-ERCC1 restricts LINE-1 retrotransposition. We focused our attention on pathway-specific adapters that segregate the nuclease activity into distinct pathways: XPA for NER and SLX4 (FANCP) for FA crosslink repair^40,41^. We generated two independent mutants of *XPA* and *SLX4* in addition to a further *XPF* line (Supplementary Fig. 3l,m and 4c,d,g,h). We found the frequency of LINE-1 integration events in *XPA*-deficient cells is indistinguishable from wild type but *SLX4*-deficient cells have increased LINE-1 integrations (Fig. 2a,b). In agreement with our findings, in three different genome-wide screens for LINE-1 regulators, XPA was not detected as a suppressor of retrotransposition^13,21,24^. However, NER has previously been shown to limit retrotransposition; it is plausible that differences in the cell lines used between these studies may explain this discrepancy^19^. These data indicate that it is the role of XPF-ERCC1 in crosslink repair that is critical to restricting LINE-1 retrotransposition.

FA DNA crosslink repair is a highly regulated process with an upstream E3-ubiquitin ligase signaling module that modifies FANCD2. Mono-ubiquitinated FANCD2 promotes incisions at sites of damaged DNA^42^. We found that the mono-ubiquitination of FANCD2 increased on DOX-induced expression of LINE-1 in K562 cells. This induction was comparable to that induced with the DNA crosslinking agent, mitomycin C (MMC). No such induction was observed in K562 cells treated with DOX that did not harbor the LINE-1 cassette (Fig. 2c).

**Extended Data Fig. 2:**
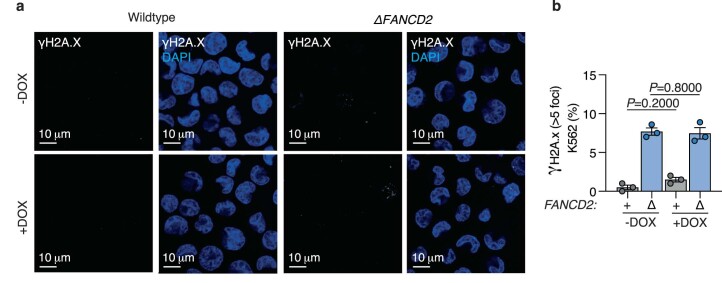
DNA damage following DOX induction in K562 wildtype cell line. **a)** Representative images of γ-H2A.x foci in wildtype or *ΔFANCD2* K562 cells (that do not contain the L1-G418^R^ reporter) before and after DOX treatment for 48 hours. Scale bar, 10 μm. **b)** Quantification of the percentage of wildtype or *ΔFANCD2* K562 cells with >5 foci, *n* = 3 independent experiments, the bar represents the mean, the error bars represent the s.e.m. and *P* values were calculated by two-tailed Mann-Whitney U-test. Source data

It has previously been shown that LINE-1 activation causes DNA DSBs, replication stress, checkpoint activation and cell cycle arrest^21,24,28^. Since the expression of LINE-1 led to the activation of crosslink repair and mono-ubiquitination of FANCD2, we hypothesized that crosslink repair may act to prevent LINE-1-induced DNA DSBs. We generated *FANCD2*-deficient K562 cells (Supplementary Fig. 4e,f), induced the expression of LINE-1 and quantified DNA damage. We measured TP53-binding protein 1 (53BP1, a surrogate marker of DNA DSBs) and phosphorylation of H2A.X (γ-H2A.X, an alternative marker of DSBs) by immunofluorescence. DOX treatment does not lead to increased DNA damage in wild-type L1-G418^R^ K562 cells (Extended Data Fig. 2a,b), but in the absence of FANCD2, an increased proportion of cells had persistent DNA damage marker foci (Fig. 2d–g). These data indicate that in the absence of crosslink repair factors, the expression of LINE-1 leads to DNA damage signaling and genome instability.

We tested whether upstream components of DNA crosslink repair limit retrotransposition. We assessed the frequency of LINE-1 integration events in cells deficient in *FANCA*, *FANCD2* or *XPF*. FANCA is a critical factor for the assembly of the FA core complex, the ubiquitin ligase responsible for FANCD2 ubiquitination. We found increased LINE-1 integration frequency for each mutant cell in both LINE-1 endonuclease deficient and proficient backgrounds (Fig. 2h,i, Supplementary Fig. 4a,b,e,f and Extended Data Fig. 1e). The frequency of LINE-1 integration events in *FANCA*-deficient cells was comparable to those in cells lacking FANCD2 or XPF.

These data indicated that the mono-ubiquitination of FANCD2 is critical to prevent LINE-1 retrotransposition. We complemented *FANCD2*-deficient cells with either wild type or mutant FANCD2 in which the mono-ubiquitinated lysine is mutated to arginine, K561R (Supplementary Fig. 4i,j)^43^. In contrast to wild type, the K561R mutant was unable to suppress retrotransposition (Fig. 2j). The K561R data strongly link FANCD2 to FA DNA crosslink repair rather than its roles outside this pathway^44,45^.

**Extended Data Fig. 3:**
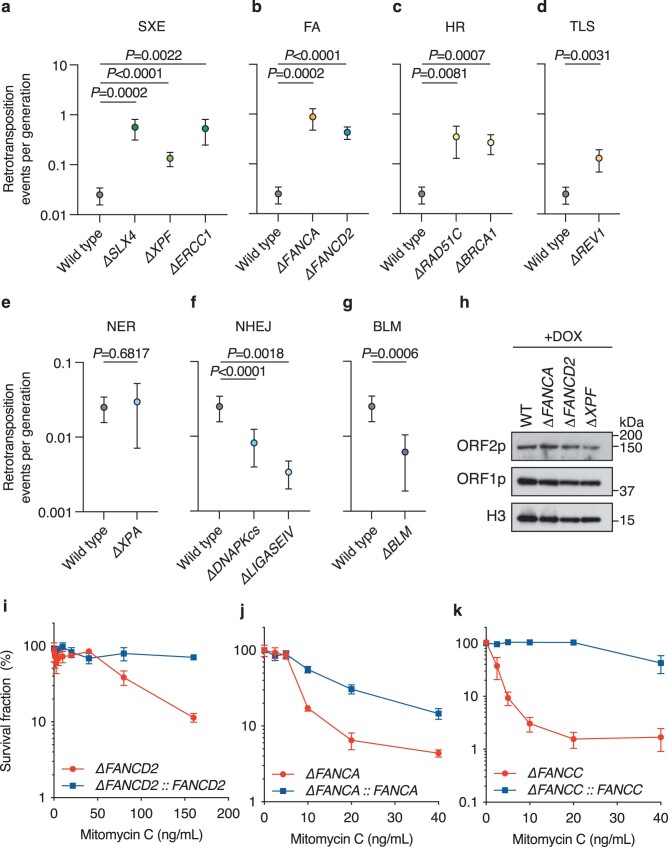
Fluctuation analysis in K562 L1-G418^R^ cell-lines, LINE-1 protein expression in mutant cell lines, and cell fitness in complemented human Fanconi fibroblasts. Independent cultures of each mutant were established from single cells and treated with DOX for 10 days. Each culture was then plated in one well of a 6-well plate containing semi-solid methylcellulose media and G418. Plating efficiency was determined for four cultures by performing three 10-fold serial dilutions and plating without G418. Statistical analysis was performed as described previously^46^. *n* = 3 independent experiments, the dot represents the mean and the error bars represent the s.e.m., *P* values were calculated by two-tailed Mann-Whitney U-test; **a)** SLX4, XPF and ERCC1 (SXE), **b)** Fanconi anemia (FA), **c)** Homologous recombination (HR), **d)** Translation synthesis (TLS), **e)** Nucleotide excision repair (NER), **f)** Non-homologous end joining (NHEJ) and **g)** Bloom syndrome helicase (BLM). **h)** Immunoblot for histone H3, LINE-1 ORF1p and LINE-1 ORF2p in wildtype, *ΔFANCA, ΔFANCD2 and ΔXPF* K562 L1-G418^R^ cells after DOX treatment; H3 loading control (representative data from 2 independent experiments). **i-k)** P220 (*ΔFANCA)* and PD331 (*ΔFANCC)* human fibroblasts were complemented by ectopically expressing FANCA and FANCC cDNA, respectively. The cellular sensitivity of these cell lines to mitomycin C was tested and compared with the previously complemented cell line PD20 (*ΔFANCD2)*; **i)** PD20 and PD20 complemented cell lines, **j)** PD220 and PD220 complemented cell lines, **k)** PD331 and PD331 complemented cell lines (data in i-k represent mean and s.e.m.; *n* = 3 independent experiments). Source data

We performed fluctuation analysis to determine the rate of retrotransposition in 12 critical mutants (Extended Data Fig. 3a–g) and confirmed the results presented above (Fig. 1d–i and 2a,b,h,i)^46^. The loss of repair factors did not alter the expression of the LINE-1 ORF1p and ORF2p proteins (Extended Data Fig. 3h). We used previously described immortalized human fibroblasts from human patients with FA in complementation groups A, C and D2 (PD220, PD331 and PD20, respectively) and their complemented lines to generalize our results beyond K562 cells (Extended Data Fig. 3i–k)^47,48^. We used a previously reported plasmid-based retrotransposition assay (Fig. 2k)^31,49^ and found that the loss of FANCA, FANCC or FANCD2 led to increased retrotransposition in patient-derived immortalized primary fibroblasts (Fig. 2l–n). These data provide strong evidence that the entire FA crosslink repair pathway—upstream signaling (core complex components), FANCD2 mono-ubiquitination and downstream incision complex (SLX4 and XPF)—are all required to suppress LINE-1 retrotransposition.

### A common pathway for ICL factors to limit retrotransposition

**Fig. 3:**
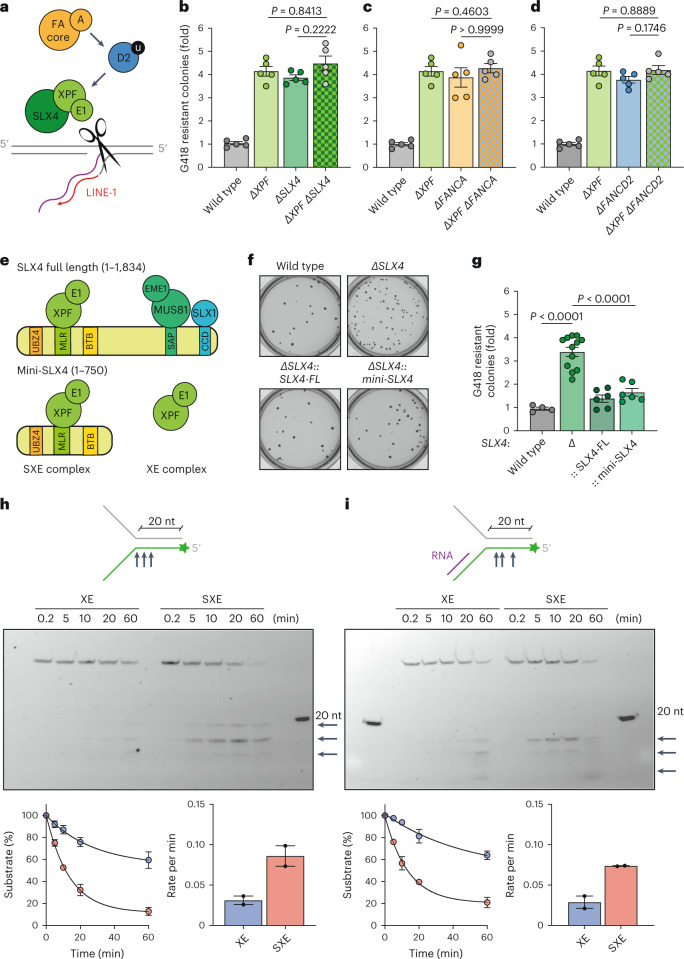
FA factors act in a common pathway to prevent retrotransposition and stimulate cleavage of putative retrotransposition intermediates. **a**, Schema illustrating the role of FA DNA ICL repair in restricting L1 retrotransposition. **b**–**d**, L1 retrotransposition measurement using L1-G418^R^ in K562 cells testing the genetic interaction between *XPF* and *SLX4* (**b**), *FANCA* (**c**) or *FANCD2* (**d**). **e**, Schema depicts the SLX4 polypeptide (1–1,834), domains and interactions with nucleases (XPF-ERCC1, MUS81-EME1 and SLX1). A truncated SLX4 1-750 (mini-SLX4) contains the region that interacts with XPF-ERCC1. **f**, Image of K562 L1-G418^R^ wild-type, SLX4 mutant and SLX4 complemented colonies grown in semisold methylcellulose media following treatment with DOX and selection with G418. **g**, L1 retrotransposition measurement using L1-G418^R^ in K562 cells (wild-type, SLX4 mutant and complementation cell lines). Each dot represents an independent experiment. **h**,**i**, XE and SXE were incubated with different fluorescent-labeled nucleic acid substrates over a time course (collecting samples at 0.2, 5, 10, 20 and 60 min). **h**, Fork-structured DNA (pseudo-3′ flap). **i**, Fork-structured DNA with an RNA–DNA hybrid. The reaction products were separated by 12% denaturing PAGE (top), and the decay of the substrate band was quantified and expressed as a percentage of the initial substrate; data were fitted using single-exponential decay (bottom, left) to calculate reaction rates (bottom, right). XE data are plotted in blue; SXE data are plotted in red, *n* = 2 independent experiments, data represent mean and s.e.m. *P* values were calculated by a two-tailed Mann–Whitney *U*-test. Source data

We hypothesized that FA crosslink repair factors act in a common pathway to limit LINE-1 retrotransposition (Fig. 3a). To test this, we generated double knockouts and assessed the frequency of retrotransposition (Supplementary Figs. 3l and 4a,e). We tested whether XPF and SLX4, its regulator in ICL repair, genetically interact and found that the frequency of integration events in the double-mutant was indistinguishable from either single mutant. This epistatic interaction suggests that both factors act together to restrict LINE-1 retrotransposition (Fig. 3b). Second, we tested the interaction between XPF and FANCA and found they were epistatic with respect to LINE-1 retrotransposition (Fig. 3c). Finally, we asked whether the downstream component and substrate of the core complex, FANCD2, genetically interacted with XPF and found a similar epistatic interaction (Fig. 3d). Together, these data strongly indicate that all these factors act in a common pathway to restrict LINE-1 activity.

### FA incision complex can cleave putative intermediates of retrotransposition

This led us to question how crosslink repair factors suppress LINE-1 retrotransposition. XPF-ERCC1 is a structure-specific endonuclease with the greatest activity on 3′ flaps and replication fork-like substrates^36,50^. SLX4 enhances the nuclease activity of XPF-ERCC1 toward replication-like structures^51^. It has been shown that a 3′ overhang is needed to prime the reverse transcription of LINE-1 (ref. ^52^). Therefore, this first intermediate, proposed in the target-primed reverse transcription (TPRT) model of LINE-1, is likely to be a 3′ flap that is the canonical substrate of SLX4-XPF-ERCC1 (refs. ^2–5^).

**Extended Data Fig. 4:**
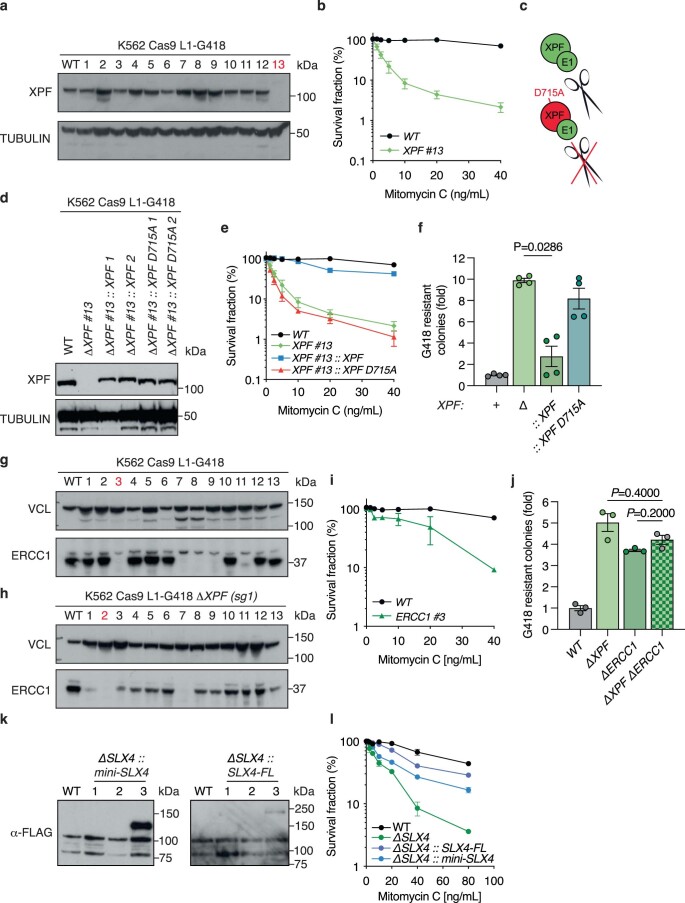
SLX4 and XPF complementation and double-knockout mutants. **a)** Immunoblot in clones targeted for XPF with sgRNA1 in wildtype or mutant K562 L1-G418^R^; TUBULIN, *α*-tubulin loading control. **b)** The cellular sensitivity of the K562 L1-G418^R^ XPF mutants to mitomycin C (*n* = 3 independent experiments). **c)** Schema depicting XPF-ERCC1 structure-specific endonuclease (top) and XPF-ERCC1 catalytically dead due to the D715A point mutation (bottom). **d)** Immunoblot for XPF in *ΔXPF#13* clone ectopically expressing either XPF or XPF(D715A) mutant cDNA. Tubulin loading control. **e)** The cellular sensitivity of the K562 L1-G418^R^ XPF mutant and complemented cell lines to mitomycin C (*n* = 3 independent experiments). **f)** L1 retrotransposition measurement using L1-G418^R^ in K562 cells (wildtype, XPF mutant and complementation cell lines), each point corresponds to an independent experiment. **g)** Immunoblot in clones targeted for ERCC1 in wildtype K562 L1-G418^R^; TUBULIN, *α*-tubulin loading control. **h)** Immunoblot in clones targeted for ERCC1 in K562 L1-G418^R^ *ΔXPF (sg1)* cell line; TUBULIN, *α*-tubulin loading control. **i)** The cellular sensitivity of the K562 L1-G418^R^ ERCC1 mutant to mitomycin C (*n* = 3 independent experiments). **j)** L1 retrotransposition measurement using L1-G418^R^ in K562 cells (wildtype, ERCC1, XPF and double-knockout cell lines), each point corresponds to an independent experiment. **k)** Immunoblot for FLAG in *ΔSLX4* clones ectopically expressing either: right, mini-SLX4 (amino acids 1–750) or left, SLX4-FL (amino acids 1-1834) cDNA. **l)** The cellular sensitivity of the SLX4 mutant and complemented cell lines to mitomycin C (*n* = 3 independent experiments). Data represent mean and s.e.m. *P* values were calculated by two-tailed Mann-Whitney U-test. Source data

While we have shown that XPF and SLX4 are epistatic with respect to retrotransposition, SLX4 does interact with two additional nucleases (MUS81 and SLX1)^53,54^. However, the loss of SLX1 or MUS81 led to negligible increase in retrotransposition (*P* = 0.0015 and *P* = 0.0003, respectively, Fig. 1h). Nevertheless, we tested whether the interaction between SLX4 and XPF-ERCC1 was sufficient to suppress retrotransposition. We complemented *SLX4*-deficient cells with either full-length SLX4 (1–1,834) or mini-SLX4 (1–750) that includes the XPF-ERCC1 binding region (MLR) but does not interact with either SLX1 or MUS81 (Fig. 3e and Extended Data Fig. 4k)^40,51,53^. Both constructs rescued hypersensitivity to crosslinking agents (Extended Data Fig. 4l) and were able to fully suppress the increase in LINE-1 retrotransposition (Fig. 3f,g). Therefore, the interaction between SLX4 and XPF-ERCC1 is critical to prevent LINE-1 retrotransposition.

Furthermore, we tested whether the nuclease activity of XPF was required to limit retrotransposition. We overexpressed wild type or catalytically dead (D715A) XPF in *XPF*-deficient cells (Extended Data Fig. 4a–e)^55,56^. Wild-type XPF suppressed the increase in retrotransposition but the catalytically dead mutant retained high levels of retrotransposition comparable to the *XPF*-null (Extended Data Fig. 4f). We generated *ERCC1*-deficient cells and found that they had elevated levels of retrotransposition (Extended Data Figs. 3a and 4g,i,j). We then generated *ΔXPFΔERCC1* double mutants and found that they were epistatic with respect to LINE-1 retrotransposition (Extended Data Fig. 4h,j).

**Extended Data Fig. 5:**
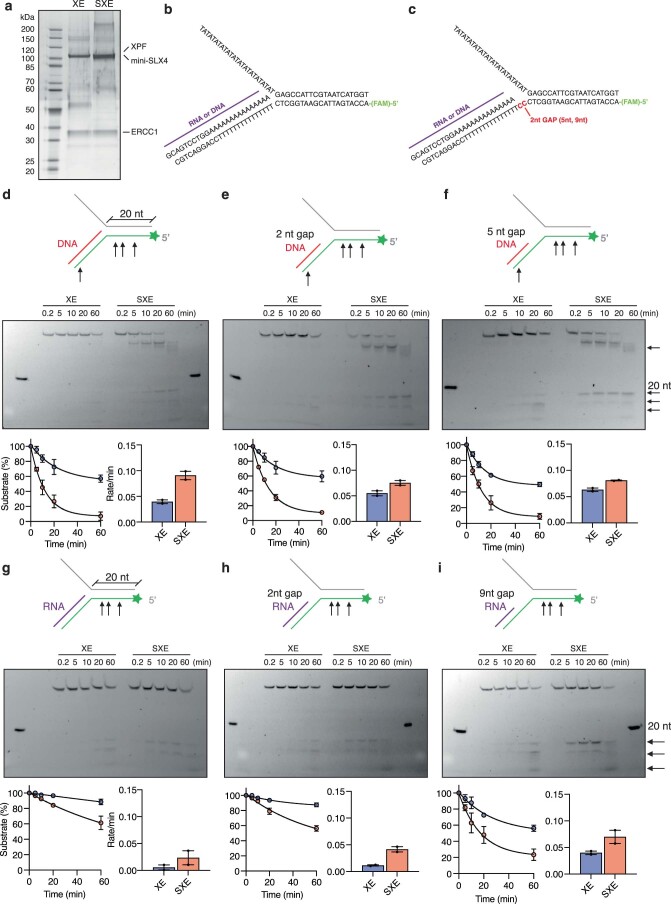
SLX4 potentiates the nuclease activity of XPF-ERCC1 on DNA:DNA and DNA:RNA substrates. **a)** Silver staining of the purified XE and SXE complexes used in the previous biochemistry assays (representative data from 2 independent experiments). **b)** Schema illustrating the substrate (annealed oligonucleotides) used in nuclease assays without a gap in the junction. **c)** Schema illustrating the substrate (annealed oligonucleotides) for nuclease assays with a gap in the junction (2, 5 or 9 nucleotides). XE and SXE were reacted with different fluorescent-labelled nucleic acid substrates, over a time course (0.2, 5, 10, 20, and 60 minutes). The reaction products were separated by 12% denaturing PAGE (top panel), and the decay of the substrate band was quantified and expressed as a percentage of the initial substrate; data were fitted using single exponential decay (bottom panel, left) in order to calculate reaction rates (bottom panel, right). XE data are plotted in blue; SXE data are plotted in red, *n* = 2 independent experiments, error bars represent s.e.m. A gap of **d)** 0, **e)** 2 or **f)** 5 nucleotides was introduced between the junction of splayed arms and the annealed DNA. A gap of **g)** 0, **h)** 2 or **i)** 9 nucleotides was introduced between the junction of splayed arms and the annealed RNA. Source data

We purified recombinant XPF-ERCC1 (XE) and mini-SLX4-XPF-ERCC1 (SXE) (Fig. 3e and Extended Data Fig. 5a) from insect cells to test whether they were able to cleave intermediates of LINE-1 retrotransposition^51^. As previously reported, we found that the activity of XE on a 3′ pseudoflap was enhanced by SLX4 (Fig. 3h). We next assessed the activity on a 3′ DNA flap with RNA annealed, designed to mimic the intermediate formed during TPRT. As a control, we also annealed DNA generating a double-stranded DNA 3′ branching structure (Extended Data Fig. 5b,c). In both cases, we found that XE cleavage was enhanced by SLX4 (Fig. 3i and Extended Data Fig. 5d–i). We also found that while both XE and SXE nicked double-stranded DNA, neither was able to nick the RNA–DNA hybrid (Extended Data Fig. 5d–i). As the length of the gap between the 3′ end of the RNA and the junction of the splayed DNA arms increased, the activity of XE and SXE was enhanced (Extended Data Fig. 5g–i). This may be due to steric hindrance akin to the inhibition of XPF-ERCC1 activity when a 3′ DNA end invades^50,57^. We have tested this on a small subset of the potential intermediates of LINE-1 retrotransposition, and SLX4-XPF-ERCC1 may show activity toward others. However, these data indicate a possible mechanism by which these factors repress LINE-1 retrotransposition. The SXE complex could cleave the 3′ DNA flap intermediate aborting integration. Akin to crosslink repair, we propose that the FA ICL repair machinery is recruited to the site of LINE-1 integration, orchestrating and promoting the incision of an intermediate of retrotransposition, aborting new integration events (Fig. 3a).

### ICL repair factors inhibit LINE-1 retrotransposition in vivo

**Fig. 4:**
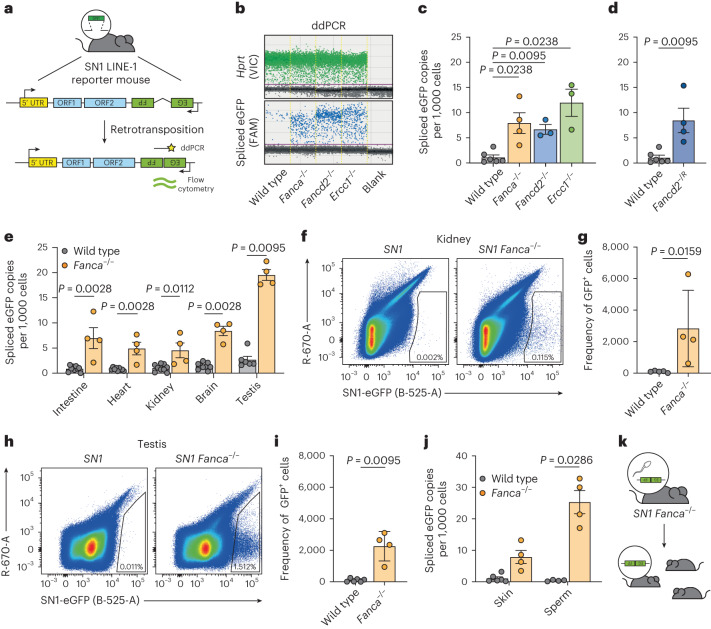
FA DNA interstrand crosslink repair is required to prevent retrotransposition in vivo. **a**, SN1 LINE-1 reporter before and after retrotransposition. ORF, open reading frame. UTR, untranslated region. **b**, Representative ddPCR plots showing frequency of spliced eGFP and *Hprt* control from skin of wild-type and mutant mice. **c**, Frequency of spliced eGFP in a skin sample of wild-type and mutant mice by ddPCR. Each point represents one mouse. **d**, Frequency of spliced eGFP in a skin sample from wild-type and *Fancd2*^*−/K559R*^ adult mice by ddPCR. Each point represents one mouse. **e**, Frequency of spliced eGFP in various tissues from wild-type and *Fanca*^*−/−*^ adult mice by ddPCR. Each point represents one mouse. **f**, Representative flow cytometry plot of kidney single-cell suspension of *SN1* and *SN1 Fanca*^*−/−*^ mice. **g**, GFP^+^ cells in kidney of *SN1* and *SN1* *Fanca*^*−/−*^ mice by flow cytometry. Each point represents one mouse. **h**, Representative flow cytometry plot of testis single-cell suspension derived from the SN1 and SN1 *Fanca*^*−/−*^ mice. **i**, GFP^+^ cells in the testis of *SN1* and *SN1* *Fanca*^*−/−*^ mice by flow cytometry. Each point represents one mouse. **j**, Frequency of spliced eGFP in epididymal sperm from wild-type and *Fanca*^*−/−*^ mice by ddPCR. Each point represents one mouse. **k**, New SN1 LINE-1 integrations in male gametes can be potentially passed onto the next generation. Data represent mean and s.e.m. *P* values were calculated by a two-tailed Mann–Whitney *U*-test. Source data

FA crosslink repair factors can coordinate cleavage of LINE-1 intermediates and limit retrotransposition in cells but it is unknown whether this is physiologically important. Tracking LINE-1 integrations in vivo is difficult due to their abundance (approximately 600,000 copies), the extremely low rate of retrotransposition and the occurrence of new integrations at different sites in different cells. Newkirk et al. developed a new LINE-1 reporter, the SN1 LINE-1 reporter, that uses the native 5′ untranslated region to drive the expression of the codon-optimized ORF1p and ORF2p, and an enhanced green fluorescent protein (eGFP) cassette in the opposite orientation interrupted by an intron to allow detection and quantification of retrotransposition events (Fig. 4a)^58,59^. This reporter has limitations, for example the codon-optimization improves expression and increases translation. However, this reporter has indistinguishable DNA methylation and RNA expression dynamics from endogenous LINE-1 elements. Furthermore, the piRNA system that regulates LINE-1 activity also regulates the activity of this reporter^59^. For these reasons, we chose to use this system for our in vivo assays.

To test the role of ICL repair factors in vivo, we used previously characterized *Fanca-*, *Fancd2-* or *Ercc1-*deficient mice^60–62^. *Fanca-* and *Fancd2-*deficient mice are models of the human disease FA with reduced frequencies of blood stem cells, increased cancer predisposition and infertility^60,63^. *Ercc1-*deficient mice have a more severe, progeroid phenotype, succumbing to liver failure^64,65^. All three mutants are infertile due to failure of the embryonic development of primordial germ cells^62^. We crossed these repair mutants with the SN1 LINE-1 reporter and quantified the frequency of spliced eGFP using a highly sensitive droplet digital (dd)PCR assay (Fig. 4a). We found that we could detect spliced eGFP copies in skin biopsies of DNA repair-proficient mice, but that the frequency was significantly increased in *Fanca*^*−/−*^, *Fancd2*^*−/−*^ or *Ercc1*^*−/−*^ adults (7.3-, 6.1- and 11.1-fold, respectively, Fig. 4b,c). We tested the requirement for the FANCD2 ubiquitination site by generating a mouse line carrying a K559R mutation (equivalent to K561R in human). Consistent with our in vitro data, we found that *Fancd2*^*−/K559R*^ mice had an elevated frequency of spliced eGFP, comparable to *Fancd2*^*−/−*^ mice (Fig. 4c,d).

**Extended Data Fig. 6:**
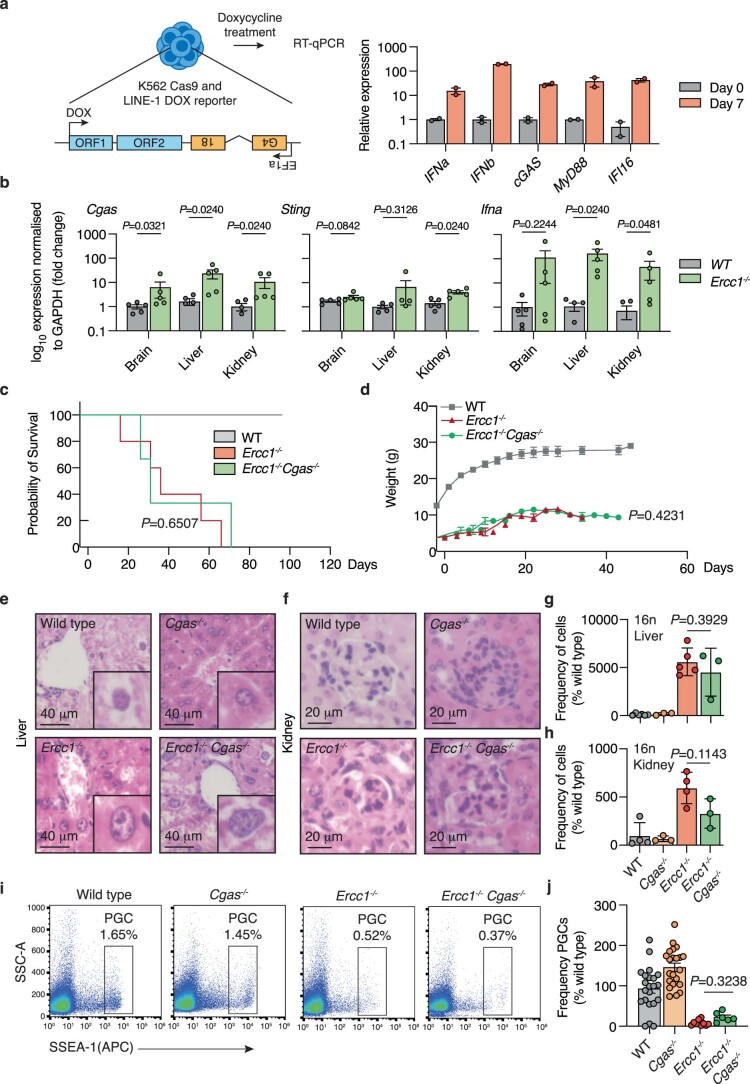
Loss of cGAS does not rescue the *Ercc1*^−/−^ phenotype. **a)** Left, schema illustrating the treatment regime in K562 L1-G418^R^ cells to assess if L1 expression induces an interferon response. Right, the expression of IFN and IFN-induced genes was assessed by RT-qPCR. *n* = 2 cultures per condition. Expression was normalized to *Gapdh* and made relative to the untreated sample. **b)** The expression of *Ifna, Cgas* and *Sting* was assessed by RT-qPCR. Each point represents one mouse. Expression was normalized to *Gapdh* and made relative to the untreated sample. **c)** Survival curve of wildtype, *Ercc1*^*−/−*^ and *Ercc1*^*−/−*^*Cgas*^*−/−*^ mice. No statistically significant difference (*P* = 0.0084) was found with the Wilcoxon test between *Ercc1*^*−/−*^ and *Ercc1*^*−/−*^*Cgas*^*−/−*^ mice. **d)** Weight curve *Ercc1*^*−/−*^ and *Ercc1*^*−/−*^*Cgas*^*−/−*^ mice (*n* = 3 per mice group). Representative histological analysis of; **e)** liver (scale bar: 40 μm) and **f)** kidney (scale bar: 20 μm) of *Ercc1*^*−/−*^*Cgas*^*−/−*^ and littermate controls. **g)** Frequency of 16n hepatocytes quantified by flow cytometry, each point represents one animal. **h)** Frequency of 16n kidney cells quantified by flow cytometry, each point represents one animal **i, j)** Frequency of SSEA1^+^ primordial germ cells in *Ercc1*^*−/−*^*Cgas*^*−/−*^ and littermate controls at E12.5 quantified by flow cytometry, each point represents one embryonic gonad. Data represent mean and s.e.m. Unless otherwise specified, *P* values were calculated by two-tailed Mann-Whitney U-test. Source data

The activation of LINE-1 can induce cGAS-STING signaling^66^. We hypothesized that cGAS-STING signaling may contribute to the phenotype of *Ercc1*^*−/−*^ mice. LINE-1 expression (L1-G418^R^) in the human K562 cell line activated cGAS-STING and interferon (Extended Data Fig. 6a). Similarly, we observed an induction of cGAS-STING and interferon responses in *Ercc1*^*−/−*^ mouse tissues (Extended Data Fig. 6b). We then generated *Ercc1*^*−/−*^*Cgas*^*−/−*^ mice to test if cGAS drove aspects of the *Ercc1*^*−/−*^ phenotype. However, we found that there was no discernible difference between *Ercc1*^*−/−*^*Cgas*^*−/−*^ and *Ercc1*^*−/−*^ mice. They had comparable longevity, growth retardation and histological defects (Extended Data Fig. 6c–j). It is therefore unlikely that cGAS-STING signaling plays a major role in the pathogenesis of the *Ercc1*^*−/−*^ mice phenotype.

**Extended Data Fig. 7:**
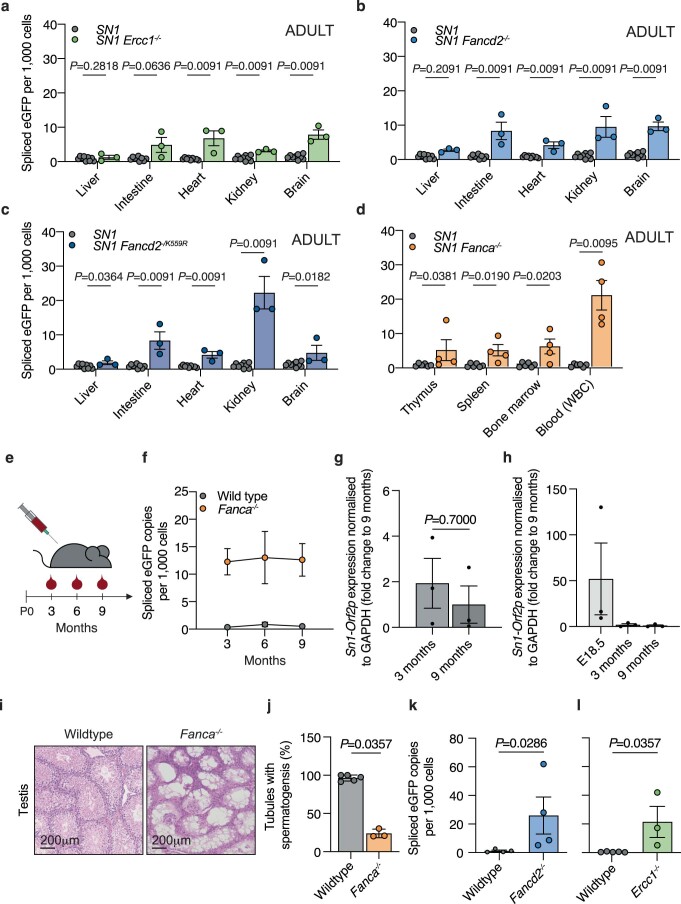
FA DNA ICL repair factors restrain SN1 LINE-1 retrotransposition. Quantification of the frequency of SN1 integrations by ddPCR in adult mice. Each point represents one mouse. Various tissues with different replication potentials were assessed in; **a)** *Ercc1*^*−/−*^, **b)** *Fancd2*^*−/−*^, **c)** *Fancd2*^*−/R*^, or **d)** *Fanca*^*−/−*^ adult mice. **e)** Schema illustrating the timing of peripheral blood harvests from wildtype and *Fanca*^*−/−*^ adult mice. **f)** Quantification of the frequency of SN1 integrations in peripheral white blood cells from the same wildtype and *Fanca*^*−/−*^ mice by ddPCR at 3, 6 and 9 months. *n* = 2 independent mice per genotype. **g)** The expression of SN1 LINE-1 reporter was quantified by RT-qPCR in peripheral white blood cells from adult mice at 6 and 9 months. Expression was normalized to *Gapdh* and made relative to the 9 months sample. *n* = 3 independent mice per genotype. **h)** Same analysis but including E18.5 liver sample. *n* = 3 independent mice per genotype. **i)** Representative histological images of the testes of wildtype or *Fanca*^*−/−*^ adult mice. Scale bar, 200 μm. **j)** Quantification of the frequency of tubules exhibiting spermatogenesis. Each point represents one mouse **k, l)** Quantification of the frequency of SN1 integrations in; **k)** testis from adult wildtype and *Fancd2*^*−/−*^ mice and **l)** fetal testis from wildtype and *Ercc1*^*−/−*^ E18.5 embryos by ddPCR. Each point represents one mouse or embryo. Data represent mean and s.e.m. Unless otherwise specified, *P* values were calculated by two-tailed Mann-Whitney U-test. Source data

**Extended Data Fig. 8:**
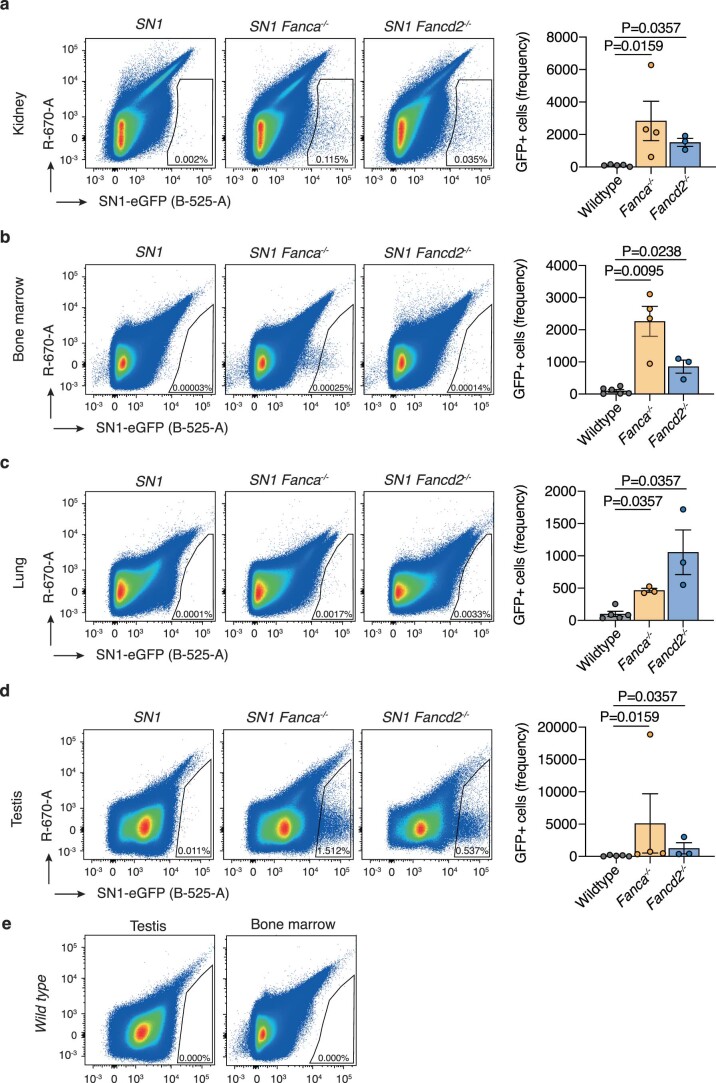
SN1 LINE-1 retrotransposition in wildtype and Fanconi mice analysed by FACS. Representative flow cytometry plot and quantification of GFP+ cells from *SN1*, *SN1* *Fanca*^*−/−*^ and *SN1* *Fancd2*^*−/−*^ mice from **a)** kidney, **b)** bone marrow, **c)** lung, **d)** testis. Each point represents one mouse, data represent mean and s.e.m. *P* values were calculated by two-tailed Mann-Whitney U-test **e)** Representative flow cytometry plot of single-cell suspension derived from bone marrow and testis of wildtype mice (that did not carry the SN1 LINE-1 reporter). Source data

We isolated DNA from different tissues of adult mice with different embryonic origins and replicative potentials and found that the loss of *Fanca* resulted in an increased frequency of spliced eGFP (Fig. 4e). We performed the same analysis on tissues from *Ercc1*^*−/−*^, *Fancd2*^*−/−*^ and *Fancd2*^*−/K559R*^ mice observing a comparable pattern of elevated spliced eGFP to wild-type controls (Extended Data Fig. 7a–d). The ddPCR approach may detect both new SN1 LINE-1 integrations but may also amplify unintegrated SN1 LINE-1 complementary DNA. We therefore asked whether we could detect eGFP protein as, in analogy to similar reporters, it is thought that expression of eGFP will only occur after retrotransposition (Fig. 4a)^29,67–70^. A single-cell suspension of the kidney was analyzed by flow cytometry. We found that in the absence of FANCA, there was a significant increase in the frequency of GFP^+^ cells (Fig. 4f,g). We extended this analysis to the bone marrow and lung (tissues from which we could generate single-cell suspensions) and again found a significant increase in the frequency of GFP^+^ cells in the absence of either FANCA or FANCD2 (Extended Data Fig. 8a–f). We could not detect GFP^+^ cells in the absence of the SN1 LINE-1 reporter (Extended Data Fig. 8i). These data show an increase in the frequency of spliced eGFP and cells expressing GFP in the absence of the FA pathway.

**Extended Data Fig. 9:**
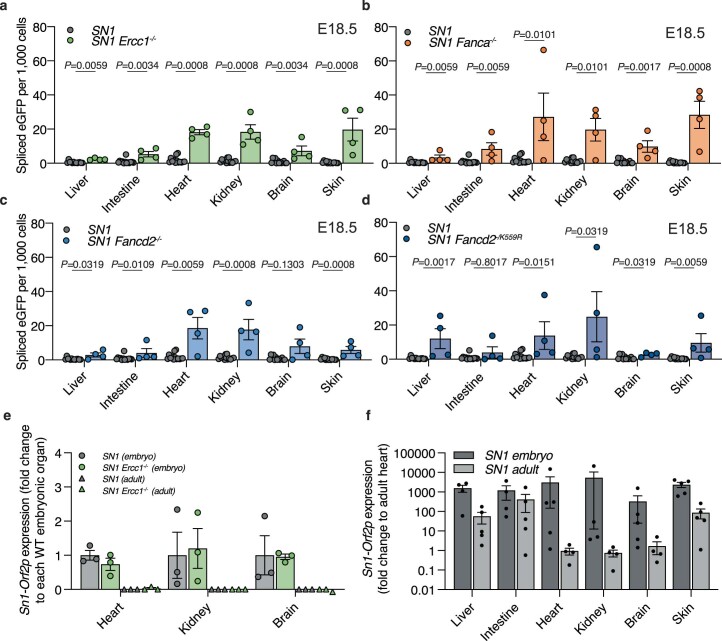
FA DNA ICL repair factors restrain SN1 LINE-1 retrotransposition during embryogenesis. Quantification of the frequency of SN1 integrations was quantified by ddPCR in E18.5 embryos. Each point represents one embryo. Various tissues with different replicative potentials were assessed in; **a)** *Ercc1*^*−/−*^, **b)** *Fanca*^*−/−*^, **c)** *Fancd2*^*−/−*^, or **d)** *Fancd2*^*-/R*^ E18.5 embryos. **e)** The expression of SN1 LINE-1 reporter *(Sn1 Line-1 Orf2p)* was quantified by RT-qPCR in multiple tissues of wildtype and *Ercc1*^*−/−*^ adult mice and embryos carrying the SN1 LINE-1 reporter. Expression was normalized to *Gapdh* and made relative to wildtype E18.5 embryos. *n* = 3 adult mice per genotype and *n* = 3 embryos per genotype **f)** The expression of SN1 LINE-1 reporter *(Sn1 Line-1 Orf2p)* was quantified by RT-qPCR in multiple tissues of wildtype adult mice and embryos carrying the SN1 LINE-1 reporter. Expression was normalized to *Gapdh* and made relative to the adult heart. Each point represents one animal. Data represent mean and s.e.m. *P* values were calculated by two-tailed Mann-Whitney U-test. Source data

We measured the messenger RNA (mRNA) of SN1 LINE-1 reporter and repair factors to determine whether expression differences could explain the increase in retrotransposition (Supplementary Fig. 6a–c). We found that the liver, intestine and skin had particularly high expression of SN1 LINE-1 (Supplementary Fig. 6b). We found similar expression of *Fanca*, *Slx4* and *Fancd2* between tissues (Supplementary Fig. 6c). We next demonstrated that the loss of these repair factors did not change the expression of the SN1 LINE-1 reporter (Extended Data Fig. 9e and Supplementary Fig. 6d). Together, these results demonstrate that crosslink repair plays an important role in suppressing retrotransposition in mice as well as in human cell lines.

If retrotransposition occurs throughout life, we predicted that the frequency of integrations should accumulate with time. We purified peripheral nucleated white blood cells from wild-type or *Fanca*^*−/−*^ 3-month-old mice and found that in the absence of *Fanca*, there was a 25-fold induction in the frequency of spliced eGFP (Extended Data Fig. 7d). We subsequently bled the same animals at 6 and 9 months (Extended Data Fig. 7e). We did not see any further increase suggesting that the role of crosslink repair in limiting retrotransposition is independent of age (Extended Data Fig. 7f). To ask whether the transcription of the reporter changes with age, we measured the expression of the reporter in the peripheral white blood cells at 3 and 9 months (Extended Data Fig. 7g). There was no significant difference between these time points. We then compared this to the fetal liver at E18.5 (a major site of hematopoiesis) and found that there was a much higher expression of the reporter (Extended Data Fig. 7h). This suggests that the reporter may be more heavily transcribed during fetal development than in adult life.

The testes of *Fanca*^*−/−*^ mice stood out as the tissue with the highest rate of retrotransposition (Fig. 4e,h,i). This was surprising as the loss of crosslink repair vastly reduces the number of germ cells with *Fanca*^*−/−*^ males having a 4.1-fold reduction in the frequency of tubules exhibiting spermatogenesis^62^. Therefore, when comparing the testes of wild-type and *Fanca*^*−/−*^ males, we analyzed tissues with different cellular compositions (Extended Data Fig. 7i,j). To circumvent this, we purified epididymal sperm, isolated genomic DNA and assessed spliced eGFP. These data showed a 60.5-fold increase in the frequency of spliced eGFP events in *Fanca*-deficient sperm when compared to littermates (Fig. 4j). Furthermore, the spliced eGFP frequency in sperm was significantly higher than in skin, suggesting that the male germline may be particularly vulnerable to LINE-1 retrotransposition. This is plausible as germ cells undergo complex epigenetic reprogramming events leading to activation of LINE-1 expression and retrotransposition^16^. We also observed increased rates of spliced eGFP in *Fancd2*^*−/−*^ adult testes and *Ercc1*^*−/−*^ fetal testes (Extended Data Fig. 7k,l and 8g,h). This is of particular importance, as LINE-1 elements that expand in the germline will be passed on to the next generation and potentially cause disease (Fig. 4k)^1,71^. Here, we identify DNA crosslink repair as a further tier of protection limiting retrotransposition in these uniquely vulnerable cells.

### Maternal and zygotic FA ICL repair factors suppress LINE-1

**Fig. 5:**
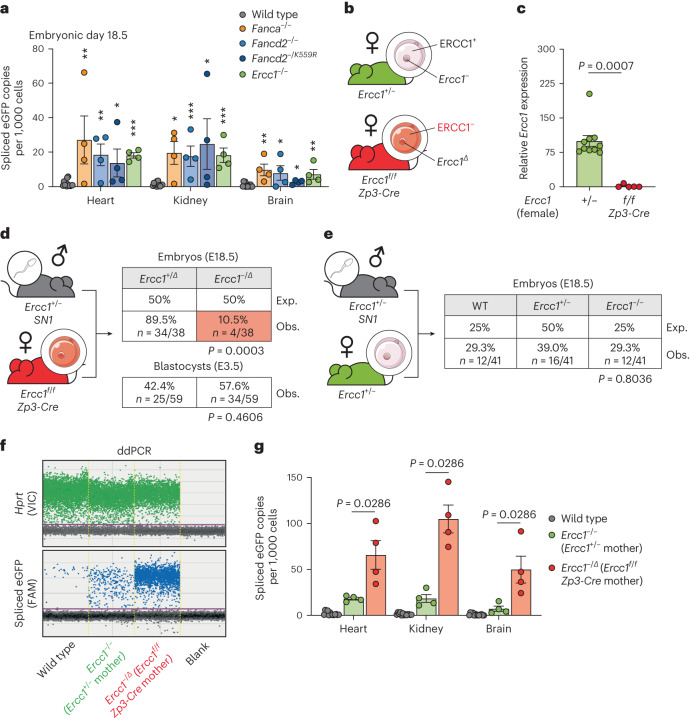
Embryonic and maternal ERCC1 limit retrotransposition during development. **a**, Quantification of frequency of spliced eGFP copies at E18.5 in wild type, *Fanca*^*−/−*^, *Fancd2*^*−/−*^, *Fancd2*^*−/K559R*^ or *Ercc1*^*−/−*^ by ddPCR. Each point represents one embryo. **b**, The strategy to collect oocytes from either *Ercc1*^*+/−*^ or *Ercc1*^*f/f*^ *Zp3-Cre* females. **c**, Expression of *Ercc1* was quantified by single-cell qPCR in oocytes. Expression was normalized to *Gapdh* and made relative to *Ercc1*^*+/−*^. Each point represents one oocyte. **d**, *Ercc1*^*−/Δ*^ embryos were assessed when born from *Ercc1*^*f/f*^*Zp3-Cre* females and *Ercc1*^*+/−*^ males. The frequencies at E18.5 (top) or E3.5 (bottom) were assessed. *P* values were calculated by a two-sided Fischer’s exact test. Exp., expected; Obs., observed. **e**, The frequency of *Ercc1*^*−/−*^ embryos was assessed at E18.5 when born from *Ercc1*^*+/−*^ female and *Ercc1*^*+/−*^ male. *P* values were calculated by a two-sided Fischer’s exact test. **f**, Representative ddPCR plots showing the frequency of spliced eGFP and *Hprt* control in E18.5 embryos from *Ercc1*^*−/−*^ or *Ercc1*^*−/Δ*^ embryos from *Ercc1*^*+/−*^ or *Ercc1*^*f/f*^*Zp3-Cre* mothers, respectively. **g**, Quantification of frequency of spliced eGFP copies at E18.5 in wild-type *Ercc1*^*−/−*^ or *Ercc1*^*−/Δ*^ tissues from *Ercc1*^*+/−*^ or *Ercc1*^*f/f*^ *Zp3-Cre* mothers, respectively. Each point represents one embryo. Data represent mean and s.e.m. Unless otherwise specified, *P* values were calculated by a two-tailed Mann–Whitney *U*-test (NS, not significant *P* > 0.05, *P ≤ 0.05, **P ≤ 0.01, ***P ≤ 0.001, ****P ≤ 0.0001). Source data

As LINE-1 integrations do not accumulate with age and are comparably increased across different somatic tissues, we hypothesized that crosslink repair factors restrict retrotransposition during embryogenesis. We assessed the frequency of spliced eGFP at day 18.5 of embryonic development (E18.5) (Fig. 5a). We found that *Ercc1*^*−/−*^, *Fanca*^*−/−*^, *Fancd2*^*−/−*^ and *Fancd2*^*−/K559R*^ embryos had an elevated frequency of spliced eGFP in multiple tissues (Fig. 5a and Extended Data Fig. 9a–d). We assessed the mRNA expression of the SN1 LINE-1 reporter at E18.5 and found no difference between wild-type and *Ercc1*^*−/−*^ embryos (Extended Data Fig. 9e). However, it was striking that the expression of the SN1 LINE-1 reporter was significantly higher in all embryonic tissues when compared to the same tissues in adults (Extended Data Fig. 9f). This suggests that the SN1 LINE-1 reporter is most transcriptionally active during embryogenesis. Taken together, this shows that DNA crosslink repair acts to limit LINE-1 retrotransposition during embryonic development.

It has previously been shown that epigenetic reprogramming results in transcriptional activation of LINE-1 in the zygote and that retrotransposition events that occur during early development lead to somatic and germline mosaicism^14,72–74^. This led us to ask whether DNA crosslink repair factors could limit integration during this period. At fertilization, the RNAs and proteins present in the zygote are maternally derived. As homozygous crosslink repair-deficient mice are infertile, we had to use a conditional allele of *Ercc1* (ref. ^62^). We crossed the conditional allele of *Ercc1* with the *Tg(Zp3-cre)*^*93Knw*^ allele in which Cre recombinase is expressed under the control of the zona pellucida 3 promoter^75^. This would allow us to generate *Ercc1*-deficient oocytes: in other words, an *ERCC1* maternal deletion^76^ (Fig. 5b). We assessed whether (1) oocytes from *Ercc1*^*+/−*^ females carried *Ercc1* mRNA and (2) if this *Ercc1* mRNA was lost in the maternal deletion (*Ercc1*^*f/f*^*;Zp3-Cre*). We performed quantitative PCR with reverse transcription (RT–qPCR) on individual oocytes harvested from either *Ercc1*^*+/−*^ or *Ercc1*^*f/f*^*;Zp3-Cre* superovulated females. First, we found that oocytes from *Ercc1*^*+/−*^ females had detectable *Ercc1* mRNA (Fig. 5c). This suggests that the *Ercc1*^*−/−*^ mutants we previously studied (Figs. 4 and 5a) had maternal *Ercc1* mRNA and protein during the first zygotic divisions. Second, this mRNA was lost in oocytes from *Ercc1*^*f/f*^*;Zp3-Cre* females showing that we are able to use this strategy to generate oocytes lacking *Ercc1* mRNA (Fig. 5c).

We next asked whether maternal *Ercc1* mRNA safeguarded the development of ERCC1-deficient zygotes. We crossed the maternal *ERCC1*-deletion females (*Ercc1*^*f/f*^*;Zp3-Cre*) with *Ercc1*^*+/−*^ males to generate maternal-zygotic *ERCC1* deleted embryos (*Ercc1*^*−/Δ*^, Fig. 5d). We harvested preimplantation embryos at E3.5 and assessed the frequency of *Ercc1*^*−/Δ*^ blastocysts. There was no significant difference between the observed and expected frequency at E3.5 (Fig. 5d). We assessed the frequency of *Ercc1*^*−/Δ*^ embryos at E18.5. The expected frequency was 50%; however, the observed frequency was 10.5% (Fig. 5d). This contrasts with *Ercc1*^*−/−*^ embryos generated from *Ercc1*^*+/−*^ intercrosses in which there was no significant difference between the expected and observed ratios (Fig. 5e). This suggests that maternal-zygotic deletion of *ERCC1* leads to a significant decrease in survival during postimplantation embryonic development.

**Extended Data Fig. 10:**
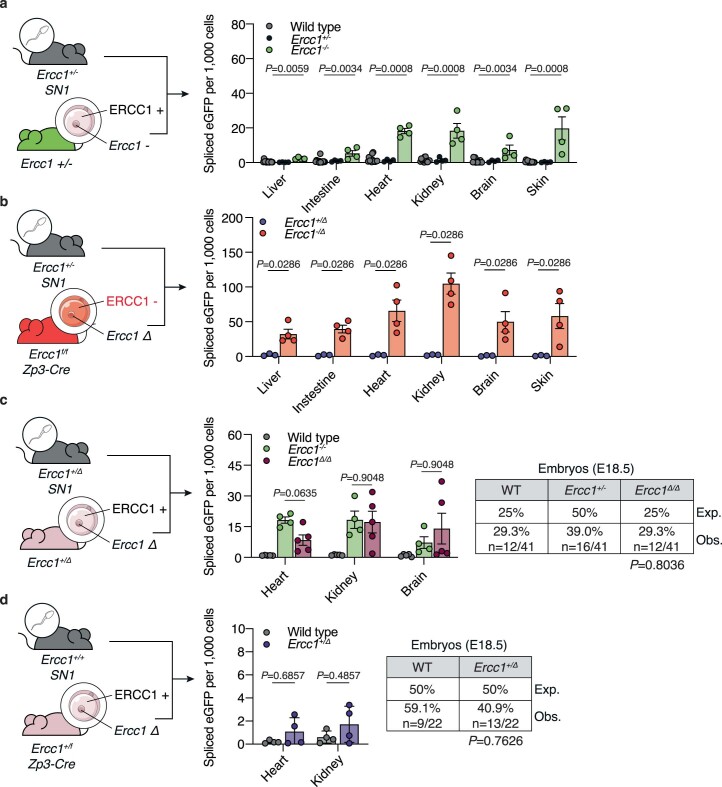
Maternal and zygotic ERCC1 contribute to LINE-1 retrotransposition restriction. **a)** Schema showing the paternal genotype, the maternal *Ercc1* genotype and the inheritance of ERCC1 in the oocyte/zygote. In this cross, maternal *Ercc1* mRNA is present in the oocyte. E18.5 embryos were obtained and frequency of spliced eGFP in multiple tissues was assessed by ddPCR. Each point represents one embryo. **b)** In this cross, maternal *Ercc1* mRNA is absent in the oocyte. E18.5 embryos were obtained and frequency of spliced eGFP in multiple tissues was assessed by ddPCR. Each point represents one embryo. **c)** In this cross, maternal *Ercc1* mRNA is present in the oocyte. E18.5 embryos were obtained and the frequency of SN1 integration in multiple tissues was assessed by ddPCR. Frequency of spliced eGFP was compared between *Ercc1*^*−/−*^ (from *Ercc1*^*+/−*^ intercrosses) and *Ercc1*^*Δ/Δ*^ embryos from *Ercc1*^*+/Δ*^ intercrosses. Each point represents one embryo. Table showing the frequency of *Ercc1*^*−/−*^ was as expected at E18.5. The *P* value was calculated by two-sided Fischer’s exact test. **d)** In this cross, maternal *Ercc1* mRNA is present in the oocyte. E18.5 embryos were obtained and spliced eGFP frequency in multiple tissues was assessed by ddPCR. Each point represents one embryo. Table showing the frequency of *Ercc1*^*−/−*^ was as expected at E18.5. The *P* value was calculated by two-sided Fischer’s exact test. Data represent mean and s.e.m. Unless otherwise specified, *P* values were calculated by two-tailed Mann-Whitney U-test. Source data

However, our primary aim was to ask if maternal *Ercc1* mRNA offers protection against LINE-1 retrotransposition. We found that *Ercc1*^*−/Δ*^ embryos generated from *Ercc1*^*f/f*^*;Zp3-Cre* females (that is, oocytes lacking *Ercc1* mRNA) harbored a striking increase in frequency of spliced eGFP (Fig. 5f, g). We measured these events at E18.5 (as at very early time points, for example E3.5, we obtained insufficient DNA to perform the analysis) allowing us to directly compare these data with that obtained for *Ercc1*^*−/−*^ embryos from *Ercc1*^*+/−*^ intercrosses (Fig. 5a). This comparison showed that, while *Ercc1*^*−/−*^ born from *Ercc1*^*+/−*^ intercrosses had a 12.6-fold induction in spliced eGFP, *Ercc1*^*−/Δ*^ embryos generated from *Ercc1*^*f/f*^*;Zp3-Cre* females had a much larger 75.5-fold induction (Fig. 5f,g and Extended Data Fig. 10a,b). We wanted to confirm that the difference observed was not due to the difference between the *Ercc1*^*−*^ and *Ercc1*^*Δ*^ alleles. We therefore generated *Ercc1*^*Δ/Δ*^, embryos from an *Ercc1*^*+/Δ*^ intercross and found that they were born at the expected ratio (Extended Data Fig. 10c, right panel). We then compared the SN1 LINE-1 spliced eGFP frequency with that observed in *Ercc1*^*−/−*^ (from *Ercc1*^*+/−*^ intercrosses) and found no difference (Extended Data Fig. 10c, left panel). Finally, we showed that *Zp3-Cre* recombinase itself did not affect either embryo survival or the frequency of SN1 spliced eGFP (Extended Data Fig. 10d). Therefore, our data reveal an important role for maternal *Ercc1* in suppressing LINE-1 retrotransposition.

Early zygotic development is extremely vulnerable as epigenetic reprogramming causes temporary transcriptional activation of LINE-1 elements resulting in new integrations that can cause disease or be passed on to the next generation. We find that both maternal and zygotic *Ercc1* play a role in preventing these LINE-1 integration events. Together, these data show that during this particularly vulnerable phase, DNA crosslink repair acts as a failsafe mechanism to prevent LINE-1 retrotransposition.

## Discussion

Several mechanisms limit LINE-1 retrotransposition but historically the role of DNA repair has been underappreciated. Landmark studies identified a role of DNA repair in regulating retrotransposition in cells^13,17–24,28–30,77–79^. We build on this, showing that FA DNA ICL repair factors act in a common pathway to restrain LINE-1 retrotransposition. Biochemically, these factors can cleave putative nucleic acid intermediates of retrotransposition, preventing the priming of reverse transcription. We show that crosslink repair factors also limit LINE-1 retrotransposition in vivo under physiological conditions. This allowed us to identify a particular dependence on crosslink repair during male germ cell and early zygotic development. Together, this shows that FA crosslink repair acts as a failsafe mechanism to limit LINE-1 retrotransposition when other mechanisms of restraint are attenuated.

We have added to the rapidly expanding list of DNA repair pathways (that is, homologous recombination and translesion synthesis) required to limit retrotransposition. It is striking that the repair processes identified have well-described roles in maintaining DNA replication and limiting the formation of DNA DSBs. These factors may act directly on replication fork intermediates or alternative DNA structures, for example DNA secondary structures, to limit retrotransposition. Further studies will be essential to explain whether these factors are redundant to each other or are each dedicated to limiting retrotransposition in distinct situations.

It is possible that in the absence of these repair factors, the expression of the LINE-1 reporter (either mRNA or protein) is increased, leading to elevated retrotransposition. However, we did not observe altered expression of ORF1p and ORF2p in the absence of FANCA, FANCD2 or XPF (Extended Data Figs. 3h and 9e and Supplementary Fig. 6d). The FA pathway regulates an incision complex (SLX4-XPF-ERCC1) that cleaves DNA at ICLs to allow lesion bypass and replication to reach completion. It was striking to us that an intermediate of the LINE-1 TPRT model is a 3′ flap: the canonical substrate for XPF-ERCC1 (ref. ^36^). Our observation that the addition of SLX4 can stimulate XPF-ERCC1 cleavage of these intermediates provides a simple mechanism to limit retrotransposition: namely preventing priming of reverse transcription. However, canonical TPRT is not the only model proposed for LINE-1 integration. It has been proposed that ORF2p may generate staggered nicks on opposite stands, generating a DSB with a 3′ overhang which could act to prime reverse transcription. It has also been suggested that the resection of telomeres could generate 3′ overhangs to start retrotransposition. It is therefore possible that the FA pathway could limit retrotransposition by cleavage of one of these DNA structures that are independent of DNA replication^32,80^. Finally, LINE-1 could exploit DSBs that are generated following DNA damage and, subsequent to resection, could lead to the generation of a 3′ flap. Indeed, it remains plausible that in the absence of DNA crosslink repair, there is an increased frequency of stalled replication forks and that these forks could be the substrate for LINE-1 retrotransposition either directly (for example, through the persistence of 3′ OH groups in lagging strand synthesis) or following endonuclease-mediated cleavage^17^.

DNA crosslink repair deficiency causes FA in humans, characterized by developmental defects, bone marrow failure and cancer predisposition^81^. There are known differences in both the biology of LINE-1 elements and embryonic development between mice and humans that are important caveats to generalizing the findings of this study. There are differences in the timing of zygote genome activation and in the piRNA system that are likely to affect LINE-1 regulation. However, it is difficult to address these differences as there are both technical and ethical difficulties that preclude studying LINE-1 retrotransposition in human physiological situations. Despite this, it is plausible that increased LINE-1 retrotransposition could contribute to the FA phenotype. LINE-1 retrotransposition events occur at differing rates in human cancers. It is striking that squamous cell carcinomas have among the highest numbers of retrotransposition events and are very common in human patients with FA^81–83^. It has been shown that squamous cell carcinomas from patients with FA shows higher levels of gene rearrangement at repetitive transposon sequences but not increased levels of retrotransposition^84^. Nonetheless, it will be interesting to test whether tumors deficient in the FA pathway have high levels of LINE-1 retrotransposition and if this contributes to carcinogenesis. Going forward, a key question will be to ask whether preventing LINE-1 transcription suppresses aspects of the FA phenotype, however, this will be technically difficult to test in vivo. It will be necessary to limit LINE-1 transcription as it could be the increased burden of LINE-1-mediated DNA damage rather than increased numbers of integration events that cause tissue dysfunction.

This study has wider implications as we identify DNA repair factors as a failsafe to prevent retrotransposition during developmental stages in which canonical mechanisms are less efficient. Germ cells and early development are particularly vulnerable to LINE-1 as physiologically necessary epigenetic reprogramming leads to loss of transcriptional silencing and activation of LINE-1 (refs. ^73,74^). As a result, de novo retrotransposition in the male germline has been shown to produce disease-causing mutant alleles^85^. We have identified DNA crosslink repair as an additional tier of protection to limit such events. It has also become clear that early development may in fact be the most critical window when retrotransposition occurs^72^. This timing allows the new insertion to be passed on to the next generation through the germline but could also lead to somatic mosaicism. We find that maternal and zygotic DNA crosslink repair factors both contribute to preventing these events. This shows that DNA repair may play a critical role during this extremely vulnerable developmental stage.

## Methods

### Cell culture

K562 cells were grown in Roswell Park Memorial Institute (RPMI) 1640 Medium (Gibco) supplemented with 10% fetal bovine serum (FBS) (Gibco) and penicillin/streptomycin and cultured at 37 °C with 5% CO_2_. Human fibroblasts were grown in Dulbecco′s modified Eagle medium supplemented with 10% FBS (Gibco) and penicillin/streptomycin, and cultured at 37 °C with 5% CO_2_. The cell lines used in the study were tested to be mycoplasma free.

### Plasmids

pB-tetO-L1-G418^R^/blast, pEAK8-FANCD2-YFP, pEAK8-FANCD2(K561R)-YFP, pJJ101/L1.3, pJJ101/L1.3(D702A) and pCEP/GFP were previously published and characterized^13,49^. pB-tetO-L1(D205A)-G418^R^/blast reporter was generated by mutagenesis from pB-tetO-L1-G418^R^/blast with Q5 Site-Directed Mutagenesis Kit Protocol (E0554; New England Laboratories). SLX4 (1–1,834) and truncation ‘mini-SLX4’ (1–750) cDNA sequences were amplified by PCR from genomic DNA and cloned into pcDNA 3.1/Zeo(+). XPF and XPF(D175A) cDNA were previously published^56^. XPF and XPF(D175A) were amplified by PCR from original plasmids and cloned into pLenti-CMV-GFP-Hygro (Addgene catalog no. 17446), where GFP was exchanged for the cDNA by Gibson assembly. FANCA and FANCC cDNAs were PCR amplified and cloned into pLenti-CMV-GFP-Hygro (Addgene catalog no. 17446), where GFP was exchanged for the cDNA by Gibson assembly. All single-guide RNAs used to generate the DNA repair genetic knockouts in K562 were cloned into pKLV2-U6gRNA5(BbsI)-PGKpuro2AmCherry-W (Addgene catalog no. 67977) or lentiGuide-Hygro-eGFP (Addgene catalog no. 99375). For constitutive Cas9 expression in K562 L1(D205A)-G418^R^ line, K562 cells were nucleofected with lentiCRISPRv2-hygro (Addgene catalog no. 98291).

### CRISPR–Cas9-mediated gene disruptions in K562 cells and cell lines generation

Guide sequences for each gene disruption can be found in Supplementary Table 1. K562 cells were nucleofected with the vector containing guides by using Lonza 2b nucleofector (T-016 program). They were either plated directly into 96-well plates as single clones with Puromycin (2.5 μg ml^−1^, Gibco) selection or 2 days posttransfection, GFP^+^ or mCherry^+^ cells were single-cell sorted into 96-well plates. After 14 days at 37 °C, individual clones were analyzed for expression of the relevant protein by western blotting, by Sanger sequencing of targeted loci CRISPR deletion and/or sensitivity to DNA damage agents. Supplementary Table 1 contains the primers used to amplify the relevant loci by PCR. PCR products were cloned into Zero Blunt TOPO (ThermoFisher) and sequenced using an SP6 oligo. To generate the K562 cell line carrying the L1 endonuclease-dead reporter, K562 cells (ATCC) were nucleofected with pB-tetO-L1(D205A)-G418^R^ and plated as single cells into 96-well plates with blasticidin (25 μg ml^−1^, Gibco) selection until colonies were visible, and then tested individual clones for integration of the plasmid with PCR in both ends (oligos in Supplementary Table 2). The K562 L1(D205A)-G418^R^ cell line was nucleofected with a plasmid containing Cas9 and plated as single cells in 96-well plates with Hygromycin B Gold (200 μg ml^−1^, InvivoGen) and after 14 days individual clones were analyzed by western blotting to confirm Cas9 expression. For FANCD2 and SLX4 complementation, K562 cells were nucleofected with the vector containing the cDNAs by using Lonza 2b nucleofector (T-016 program). They were plated into 96-well plates as single clones with selection. After 14 days, individual clones were analyzed for expression of the relevant protein by western blotting and by sensitivity to MMC. For XPF complementation, K562 cells were lentivirally transduced with the vector containing cDNAs and 48 h later put in antibiotic selection. Cells were analyzed by western blotting and by sensitivity to MMC. For FANCA and FANCC complementation, human fibroblasts (PD20 and PD331) were lentivirally transduced with the vector containing cDNAs and 48 h later put in antibiotic selection. Cells were analyzed by western blotting and by sensitivity to MMC.

### Cell viability assays

Cell viability assays in K562 cells and in human fibroblasts were performed in 96-well flat-bottom plates by plating 1,000 cells per well and culturing them with increasing concentrations of MMC, bleomycin, olaparib and methyl methanesulfonate, or exposing them at ultraviolet irradiation. After 4 days, MTS cell viability reagent (CellTiter 96 AQueous One Solution Cell Proliferation Assay, Promega) was added and plates were incubated at 37 °C for 4 h; absorbance at 492 nm was measured.

### L1-G418 retrotransposition assay in K562 cells

Cells were DOX-induced (1 μg ml^−1^, Sigma-Aldrich, D9891) for 10 days, maintaining 500,000 cells per ml and refreshing the DOX. After DOX induction, cells were recovered in RPMI medium for 24 h and put into six-well plates with semisolid methylcellulose containing the G418 selection (1 mg ml^−1^, Formedium Ltd, G4185). G418-resistant colonies were counted after 14 days.

### L1-G418 retrotransposition assay in human fibroblasts

Here, 80,000 cells were plated in 100 mm culture dishes and transfected the following day with 10 μl of FuGENE 6 (Promega) and 4 μg of plasmid DNA in OptiMEM medium (ThermoFisher) following the manufacturer’s instructions. PD20 (*FANCD2*^−/−^), PD220 (*FANCA*^−/−^) and PD331 (*FANCC*^−/−^) cells were transfected with pJJ101/L1.3, pJJ101/L1.3-D702A and a plasmid containing blasticidin resistance. After 24 h, fresh media was added and replenished every other day. Then, 5 days posttransfection, cells were selected with 10 μg ml^−1^ blasticidin (ThermoFisher) for 10 days, with media change every 3 days. Colonies were fixed and stained with crystal violet and colonies counted. Cells were transfected also with pCEP4/GFP to determine the transfection efficiency.

### Fluctuation analysis

K562 L1-G418^R^ wild-type and mutant lines underwent fluctuation analysis. For each line, 18 independent cultures (with cultures to determine plating efficiency) were established by plating single cells in 96-well plates in RPMI media and 1 μg ml^−1^ of DOX (replenished every 72 h). After 10 days, each culture was transferred to a single well in a six-well plate containing semisolid methylcellulose media and 1 mg ml^−1^ G418. For plating efficiency cultures, serial dilutions were prepared and plated without G418. After 10 days, colonies were counted. Analysis was carried out using http://shinyflan.its.manchester.ac.uk/ and the statistical framework described previously^46^.

### Immunoblotting

Western blots were performed as described previously^86^. The following primary antibodies were used: anti-XPF (1:1,000, D3G8C, Cell Signaling); anti-ERCC1 (1:100, sc-17809, Santa Cruz Biotechnology); anti-FANCA (1:1,000, D1L2Z, 14657, Cell Signaling Technology); anti-XPA (1:1,000, D9U5U, Cell Signaling); anti-FEN1 (1:2,000, Abcam ab109132); anti-SNM1A (1:500, Abcam ab14805); anti-SNM1B (1:500, Proteintech 13203-1-AP); anti-SLX1 (1:120, MRC PPU S701B); anti-XRCC2 (1:500, Proteintech 20285-1-AP), anti-XRCC3 (1:600, Proteintech 18494-1-AP); anti-Cas9 (1:1,000, 7A9-3A3 14697, Cell Signaling); anti-FLAG (1:200, M2 clone, F1804, Sigma-Aldrich); anti-LAMIN B1 (1:500, ab16048, Abcam); anti-α-TUBULIN (1:3,000, T6199, Sigma-Aldrich); anti-β-ACTIN (1:3,000, Abcam ab8227) and anti-VINCULIN (1:2,000, Abcam ab129002: Abcam); anti-histone H3 (1:7500, catalog no. ab1791, Abcam); anti-LINE-1 ORF1p (1:1,000, clone 4H1, catalog no. MABC1152, Merck); anti-LINE-1 ORF1p (1:1,000, MT49, a gift from K. Burns). They were used for western immunoblotting, diluted in 5% w/v BSA, 0.1% Tween-20 TBS and incubated at 4 °C overnight with gentle agitation. Secondary antibodies were diluted in 5% w/v BSA, 0.1% Tween-20 TBS and incubated for 1 h at room temperature with gentle agitation. For FANCD2 detection, cells were treated with 500 ng ml^−1^ MMC overnight and protein samples run on a 3–8% Tris-Acetate gel (ThermoFisher). Anti-FANCD2 polyclonal antisera^87^ was used.

Assessment of DNA damage markers by immunofluorescence was performed as described previously^86^. DOX treated (48 h) and untreated K562 cells were seeded on top of poly-l-lysine coverslips and spun down for 5 min. Cells were washed twice for 5 min with PBS supplemented with 500 μM MgCl_2_ and 0.5 μM CaCl_2_ (PBS-S) then fixed with 4% paraformaldehyde (43368, Alfa Aesar) for 20 min and washed with PBS-S twice for 5 min. They were then washed three times in PBS, 1% w/v Triton X-100 for 15 min. Slides were blocked in PBS, 1% w/v BSA, 1% w/v Triton X-100 for 30 min at room temperature before being incubated overnight at 4 °C with the following primary antibodies diluted in blocking buffer: antiphospho-Histone H2A.X (Ser139) (1:1,000, JBW301, Millipore); anti-53BP1 (1:1,000, NB100-304, Novus). Slides were washed three times in PBS, 1% w/v Triton X-100 for 5 m and incubated with the following secondary antibodies diluted in blocking buffer for 1 h at room temperature: antimouse Alexa Fluor 488 (1:1,000, ThermoFisher) and antirabbit Alexa Fluor 594 (1:1,000, ThermoFisher). The slides were washed three times in PBS, 1% w/v Triton X-100 for 5 min and stained with 0.5 μg ml^−1^ DAPI diluted in PBS for 10 min. Slides were washed once in water and mounted with ProLong Gold anti-fade reagent (P36934, Molecular Probes) and coverslips were placed onto slides. Images were captured using a Zeiss LSM 780 confocal microscope and analyzed with ImageJv2.9.0/1.53t and Fiji^88^. DNA damage foci per nucleus were scored blindly.

### Expression and purification of protein complexes

SF-9 insect cells were obtained from Merck (89070101-1VL) and 2 l of insect Sf9 cells were infected at 1–2 × 10^6^ cells per ml with tertiary recombinant baculovirus. They were grown for 68 h and collected. XE and SXE purification steps were carried out in NENT buffer supplemented with 20 mM Tris pH 8.0, 5 mM TCEP, 150-400 mM NaCl, 10% glycerol and protein inhibitors cocktail. Cells were homogenized in NENT buffer supplemented with 40 M imidazole pH 8.0 and 0.1% NP-40 followed by nickel affinity chromatography on NTA agarose (Qiagen). Proteins were eluted with NENT buffer containing 250 mM imidazole pH 8.0. For SXE complex, an maltose-binding protein affinity step (NEB, E8022L) was included and the complex was eluted with 20 mM maltose. The maltose-binding protein-tag was cleaved with tobacco etch virus protease O/N at 4 °C. Complexes were diluted with NENT buffer to reduce salt to 200 mM NaCl and loaded on HP Heparin column (GE Healthcare) and eluted with a salt gradient. Concentrated samples were purified on HiLoad Superose 6 (GE Healthcare) and combined fractions were flash-frozen in liquid N_2_.

### Nuclease assays

All reactions were carried out in nuclease buffer: 10–50 mM Tris (pH 8.0), 50 mM NaCl, 2 mM MgCl_2_, 1 mM TCEP (Tris(2-carboxyethyl) phosphine–HCl) solution (Pierce, catalog no. 77730) (0.5 M TCEP, pH 7.0), 0–5% glycerol and 0.1 mg ml^−1^ BSA (NEB) at 22 °C. Oligonucleotides were labeled with FAM on the 5′ terminus as shown in Fig. 3h,i and Extended Data Fig. 5b,c. Oligos sequences are shown in Supplementary Table 3 and Extended Data Fig. 5b,c. Substrates were purified on 15% denaturing PAGE gel, desalted and annealed by slow cooling from 90 °C. Reactions were initiated by the generation of an equimolar mixture (100 nM) of the given substrate and enzyme XE and SXE. 0.2-, 5-, 10-, 20- and 60-min timepoint reactions were collected and quenched with 80% formamide, 200 mM NaCl, 10 mM EDTA and 0.01% bromophenol blue, and analyzed on 12% denaturing PAGE (1× Tris-borate-EDTA, 7 M urea, 12% 19:1 acrylamide/bis-acrylamide). Gels were scanned by Typhoon PhosphoImager (GE Healthcare). Band intensities were determined using Fiji (ImageJ). Relative substrate depletion was plotted against time and fitted by single-exponential decays using GraphPad Prism v.9. The rates of substrate depletion were plotted into a bar chart to underline the rate enhancement.

### Mice

All animal experiments undertaken in this study were approved by the Medical Research Council’s Laboratory of Molecular Biology animal welfare and ethical review body and the UK Home Office under the Animal (Scientific Procedures) Act 1986 (license no. PP6752216). Mouse husbandry was performed as described previously^62^. Mice were maintained under specific pathogen-free conditions in individually ventilated cages (GM500; Techniplast) on Lignocel FS-14 spruce bedding (IPS) with environmental enrichment at 19–23 °C with light from 07:00 to 19:00 and humidity of 45–65%, and were fed Dietex CRM pellets (Special Diet Services) ad libitum. No animals were wild and no field-collected samples were used. Mice were maintained on a C57BL/6J background. Embryos were used at E3.5 or E18.5 as indicated in the text. Samples were collected from animals at 8–12 weeks as specified in the text. Females used in timed mating experiments were aged between 6 and 18 weeks. The investigators were blinded to the genotypes of animals throughout the study and data were acquired by relying purely on identification numbers. *Fanca*^*tm1a(EUCOMM)Wtsi*^ (MGI ID 4434431), *Fancd2*^*tm1Hou*^ (MGI ID 2673422), *Ercc1*^*tm1a(KOMP)Wtsi*^ (MGI ID 4362172)*, Tnr*^*Tg(L1-EGFP)SN1Fhg*^ (MGI ID 244330), *Tg(Zp3-cre)*^*93Knw*^ (MGI 2176187) and *Cgas*^*tm1d(EUCOMM)Hmgu*^ (MGI ID 2442261) alleles have been described previously^59–62,75,89^.

### Embryo isolation

Timed matings were performed as described previously^62^. Pregnant mice were killed by cervical dislocation at E12.5 or E18.5 and embryos collected. Embryos were dissected and tissues collected and stored at −80 °C. Individual fetal gonads were placed into ice-cold PBS and quantification was performed immediately.

### Superovulation of females to obtain oocytes

Females were superovulated by treatment with 0.1 ml of PMS, after 48 h 0.1 ml of hCG and 24 h later oocytes were collected. For blastocyst collection, females were superovulated by treatment with 0.1 ml of PMS, after 48 h, 0.1 ml of hCG and mated with males. Mice were checked for the presence of a copulation plug. At E3.5, pregnant females were killed by cervical dislocation and blastocysts collected.

### Genomic DNA extraction from mouse tissues

Genomic DNA was isolated from adult mice and E18.5 embryonic tissues with Gentra Puregene Tissue (Qiagen) following the manufacturer’s instructions.

### ddPCR

All reactions contained roughly 60 ng of genomic DNA per 20 μl reactions. Probes and oligos were diluted to achieve a final concentration of 250 and 900 nM, respectively. NcoI-HF (New England Biolabs) was incorporated into the reaction and samples were incubated at 37 °C for 15 min (6.4 units of enzyme per reaction). An eight-well Bio-Rad DG8 Droplet Generator cassette was used to generate the droplets, loading 20 μl of sample and 70 μl of droplet oil. Then, 35 μl were transferred to ddPCR 96-well plate (Bio-Rad) and thermal cycling conditions were set up as follows: 95 °C for 10 min, 40 cycles of 94 °C for 30 s and 60 °C for 1 min, 98 °C for 10 min. Samples were analyzed in Bio-Rad QX200 Droplet Reader. Results were analyzed using Bio-Rad QuantaSoft software. All experiments were performed using FAM and VIC-labeled probes. Oligos and probes in Supplementary Table 2.

### Quantification of GFP positive cells in SN1 mice

Kidney, lung, femur and testes were isolated from adult mice and placed in cold PBS. To obtain single-cell suspensions from kidney, the organ was chopped into small pieces in Petri dishes and pipetted up and down with PBS. The tissue was recovered from the bottom of the tube and treated in 5 ml of Hank’s buffered saline solution (HBSS) containing 25 mg collagenase II at 37 °C for 45 min. After that, the tissue was filtered through a 70 μm cell strainer, spun down and resuspended in fluorescence-activated cell sorting (FACS) buffer (PBS/2.5% v/v FBS). For lung single-cell suspension, procedure was identical but pieces of tissue were treated with HBSS containing 25 mg collagenase II and 10 μg ml^−1^ DNase for 1 h. For bone marrow, cells were isolated from tibiae and femurs with FACS buffer and strained through 70 μm cell strainers. Testes were placed in a 100 mm culture dish containing 10 ml of PBS. Testicular tubules were separated from tunica albuginea and dissociated with forceps. Tubules were transferred to 15 ml conical tube containing 5 ml of HBSS with 500 μl of 5 mg ml^−1^ collagenase IV and 25 μl of 10 mg ml^−1^ DNase solution. This was incubated for 10 min at 37 °C. Supernatant was discarded and tubules were collected and passed to another conical tube containing 5 ml of HBSS with 500 μl of 5 mg ml^−1^ collagenase IV, 25 μl of 10 mg ml^−1^ DNase solution and 25 μl of 10 mg ml^−1^ hyaluronidase. This was incubated for 10 min at 37 °C, shaking the tube every 2 min. Cells were filtered in a 70 μm strainer, spun down and resuspended in FACS buffer. Single-cell suspensions were analyzed by flow cytometry on LSRII (BD Bioscience) with data acquired in FACSDivav6.5 (BD) and analyzed on FlowJo v.10.1r5 (FlowJo LLC) to calculate the frequency of GFP^+^ cells.

### RT–qPCR and gene expression analysis

Total RNA was extracted from either K562 cells or adult mice and embryonic tissues using RNAeasy Kit (Qiagen) and first-strand cDNA was synthesized using Quantitect Reverse Transcription Kit For cDNA Synthesis (Qiagen) following the manufacturer’s instructions. Total RNA was extracted and cDNA was synthesized from mouse oocytes using Single Cell-to-CT RT–qPCR Kit (ThermoFisher). Real-time qPCR analysis for expression of interferons and interferon-inducible genes in K562 cells and in mouse tissues (oligos in Supplementary Table 2) was performed using Brilliant II SYBR Green QPCR Master Mix (catalog no. 600828, Agilent Technologies) in a Viia7 (ThermoFisher) cycler at 50 °C for 2 min, 95 °C for 10 min and 40 cycles of 95 °C for 15 s and 60 °C for 1 min. Mean threshold cycles were determined from three technical repeats using the comparative *C*_T_ method. All expression levels were normalized to human *GAPDH* or mouse *Gapdh* (oligos can be found in Supplementary Table 2). *Orf2p Sn1 Line-1* expression (oligos in Supplementary Table 2) and DNA repair factors expression in adult and embryonic mice tissues was measured using TaqMan Fast Advance Gene Expression Master Mix (ThermoFisher) in a Viia7 (ThermoFisher) cycler at 95 °C for 20 s and 40 cycles of 95 °C for 1 s and 60 °C for 20 s. Mean threshold cycles were determined from three technical repeats using the comparative *C*_T_ method. All expression levels were normalized to mouse *Gapdh* (4352339E, ThermoFisher). *Ercc1* expression was assessed with m00468337_m1 and m00468336_g1, *Fanca* with Mm01243361_g1 and *Slx4* with Mm01342461 probes (ThermoFisher).

### Histology

Histological analysis was carried out on tissues that had been fixed in buffered formalin for 24 h. The samples were paraffin-embedded and 4 μm sections were cut before staining with hematoxylin and eosin.

### Quantification of primordial germ cells in vivo

Quantification was performed as described previously^62^. Urogenital ridges of E12.5 embryos were isolated and placed into 150 μl of trypsin solution (2.5 μg ml^−1^ trypsin (Gibco), 25 mM Tris, 120 mM NaCl, 25 mM KCl, 25 mM KH_2_PO_4_, 25 mM glucose, 25 mM EDTA, pH 7.6) and incubated for 10 min at 37 °C. Next, 1 μl of Benzonase (Millipore) was added, followed by disaggregation of the sample by pipetting and incubation for for 5 min at 37 °C. Trypsin was inactivated by adding 1 ml of PBS/5% v/v FBS. Following 10 min of centrifugation at 1,000*g*, the cell pellet was resuspended in 100 μl of Alexa Fluor 647-conjugated anti-human/mouse SSEA-1 antibody (catalog no. MC-480; BioLegend) diluted 1:100 in staining buffer (PBS/2.5% v/v FBS) and incubated at room temperature for 10 min; 300 μl of staining buffer were added to the cell suspension and samples immediately run on an ECLIPSE analyzer (Sony Biotechnology) and data analyzed using FlowJo v.10.1r5 (FlowJo LLC).

### Statistics and reproducibility

The number of independent biological samples and technical repeats (*n*) is indicated in the figure legends. Unless otherwise stated, data are shown as the mean ± s.e.m. The nonparametric Mann–Whitney *U*-test was used to determine statistical significance unless otherwise indicated in the figure legends. Analysis was performed in GraphPad Prism v.9.

### Reporting summary

Further information on research design is available in the Nature Portfolio Reporting Summary linked to this article.

## Online content

Any methods, additional references, Nature Portfolio reporting summaries, source data, extended data, supplementary information, acknowledgements, peer review information; details of author contributions and competing interests; and statements of data and code availability are available at 10.1038/s41594-023-01067-8.

### Supplementary information

Supplementary Information Supplementary Figs. 1–8.

Reporting Summary

Supplementary Tables Supplementary Tables 1–3.

Supplementary Data Source data for Supplementary Figures.

### Source data

Source Data Fig. 1 Statistical source data.

Source Data Fig. 2 Statistical source data.

Source Data Fig. 2 Unprocessed western blots.

Source Data Fig. 3 Statistical source data.

Source Data Fig. 4 Statistical source data.

Source Data Fig. 5 Statistical source data.

Source Data Extended Data Fig. 1 Statistical source data.

Source Data Extended Data Fig. 1 Unprocessed western blots.

Source Data Extended Data Fig. 2 Statistical source data.

Source Data Extended Data Fig. 3 Statistical source data.

Source Data Extended Data Fig. 3 Unprocessed western blots.

Source Data Extended Data Fig. 4 Statistical source data.

Source Data Extended Data Fig. 4 Unprocessed western blots.

Source Data Extended Data Fig. 5 Statistical source data.

Source Data Extended Data Fig. 6 Statistical source data.

Source Data Extended Data Fig. 7 Statistical source data.

Source Data Extended Data Fig. 8 Statistical source data.

Source Data Extended Data Fig. 9 Statistical source data.

Source Data Extended Data Fig. 10 Statistical source data.

## Data Availability

All data supporting the findings of this study are available within the paper and its Supplementary Information. Source data are provided with this paper.
