# Supplementary material for: Fanconi anemia DNA crosslink repair factors protect against LINE-1 retrotransposition during mouse development
Source: Nat Struct Mol Biol. 2023 Aug 14;30(10):1434–45. doi: 10.1038/s41594-023-01067-8 (PMC10584689; doi:10.1038/s41594-023-01067-8)
Supplement: Supplementary file 2 — Reporting Summary [file 41594_2023_1067_MOESM2_ESM.pdf]

nature portfolio

Corresponding author(s): Crossan

Last updated by author(s): 18 May 2023

Reporting Summary

Nature Portfolio wishes to improve the reproducibility of the work that we publish. This form provides structure for consistency and transparency in reporting. For further information on Nature Portfolio policies, see our Editorial Policies and the Editorial Policy Checklist.

Statistics

For all statistical analyses, confirm that the following items are present in the figure legend, table legend, main text, or Methods section.

n/a	Confirmed	
☐	☒	The exact sample size (*n*) for each experimental group/condition, given as a discrete number and unit of measurement
☐	☒	A statement on whether measurements were taken from distinct samples or whether the same sample was measured repeatedly
☐	☒	The statistical test(s) used AND whether they are one- or two-sided<br>*Only common tests should be described solely by name; describe more complex techniques in the Methods section.*
☐	☒	A description of all covariates tested
☐	☒	A description of any assumptions or corrections, such as tests of normality and adjustment for multiple comparisons
☐	☒	A full description of the statistical parameters including central tendency (e.g. means) or other basic estimates (e.g. regression coefficient) AND variation (e.g. standard deviation) or associated estimates of uncertainty (e.g. confidence intervals)
☐	☒	For null hypothesis testing, the test statistic (e.g. *F*, *t*, *r*) with confidence intervals, effect sizes, degrees of freedom and *P* value noted<br>*Give P values as exact values whenever suitable.*
☒	☐	For Bayesian analysis, information on the choice of priors and Markov chain Monte Carlo settings
☒	☐	For hierarchical and complex designs, identification of the appropriate level for tests and full reporting of outcomes
☒	☐	Estimates of effect sizes (e.g. Cohen's *d*, Pearson's *r*), indicating how they were calculated

Our web collection on statistics for biologists contains articles on many of the points above.

Software and code

Policy information about availability of computer code

Data collection	Flow cytometry in FACSDivav6.5 (BD)
Data analysis	Flow cytometry was on FlowJo v10.1r5 (FlowJo LLC), Data was analysed inGraphPad Prism v9, Immunofluoresence was analysed with ImageJv2.9.0/1.53t and Fiji

For manuscripts utilizing custom algorithms or software that are central to the research but not yet described in published literature, software must be made available to editors and reviewers. We strongly encourage code deposition in a community repository (e.g. GitHub). See the Nature Portfolio guidelines for submitting code & software for further information.

Data

Policy information about availability of data

All manuscripts must include a data availability statement. This statement should provide the following information, where applicable:

- Accession codes, unique identifiers, or web links for publicly available datasets
- A description of any restrictions on data availability
- For clinical datasets or third party data, please ensure that the statement adheres to our policy

All data supporting the findings of this study are available within the paper and its Supplementary Information.

Research involving human participants, their data, or biological material

Policy information about studies with human participants or human data. See also policy information about sex, gender (identity/presentation), and sexual orientation and race, ethnicity and racism.

Reporting on sex and gender	N/A
Reporting on race, ethnicity, or other socially relevant groupings	N/A
Population characteristics	N/A
Recruitment	N/A
Ethics oversight	N/A

Note that full information on the approval of the study protocol must also be provided in the manuscript.

Field-specific reporting

Please select the one below that is the best fit for your research. If you are not sure, read the appropriate sections before making your selection.

☒ Life sciences ☐ Behavioural & social sciences ☐ Ecological, evolutionary & environmental sciences

For a reference copy of the document with all sections, see nature.com/documents/nr-reporting-summary-flat.pdf

Life sciences study design

All studies must disclose on these points even when the disclosure is negative.

Sample size	For the analysis of Mendelian segregation of alleles, samples size was determined by power analysis using the following site http://biomath.info/power/chsq1gp.htm. Sufficient animals were used in order to detect a 50% reduction in expected frequency, using power of 0.8 and alpha 0.05. Others samples sizes were not predetermined as the magnitude of effect was unknown prior to the experiment.<br>For cell based retrotranspositon assays sample sizes were based upon previous publications (PMID 21940498, 8945518)<br>For in vivo retrotranspostion assays sample sizes were based upon previous publications (PMID 34083437,37126510,28630288)
Data exclusions	No data were excluded from analyses.
Replication	All experiments were repeated as described in the figure legends or materials and methods but on all occasions each experiment was performed at least twice and for each experiment all attempts of replication were successful. Different biological replicates were performed on separated days, with samples collected from separated cell culture batches or independent biological samples collected from different animals on different days and processed on different days. Samples were allocated to groups based upon subsequent genotyping. Experiments were reproducible across all repeats. All replication data are included and confirm the original findings.
Randomization	No randomization was involved as groups are based upon the genotype of the animal/emrbyo or cell line.
Blinding	The investigators were blind to the genotypes of mice and relied solely on identification numbers throughout the study. In cellular assays investigators were not blinded

Reporting for specific materials, systems and methods

We require information from authors about some types of materials, experimental systems and methods used in many studies. Here, indicate whether each material, system or method listed is relevant to your study. If you are not sure if a list item applies to your research, read the appropriate section before selecting a response.

April 2023

Materials & experimental systems

n/a	Involved in the study
☐	☒ Antibodies
☐	☒ Eukaryotic cell lines
☒	☐ Palaeontology and archaeology
☐	☒ Animals and other organisms
☒	☐ Clinical data
☒	☐ Dual use research of concern
☒	☐ Plants

Methods

n/a	Involved in the study
☒	☐ ChIP-seq
☐	☒ Flow cytometry
☒	☐ MRI-based neuroimaging

Antibodies

Antibodies used

anti-XPF (1:1,000, D3G8C, Cell Signalling); anti-ERCC1 (D10, 1:100, sc-17809, Santa Cruz Biotechnology); anti-FANCA (1:1,000, D1L2Z, 14657, Cell Signaling Technology); anti-XPA (1:1,000, D9U5U, Cell Signalling); anti-FEN1 (1:2,000, Abcam ab109132); anti-SNM1A (1:500, Abcam ab14805); anti-SNM1B (1:500, Proteintech 13203-1-AP); anti-SLX1 (1:120, MRC PPU S701B); anti-XRCC2 (1:500, Proteintech 20285-1-AP), anti-XRCC3 (1:600, Proteintech 18494-1-AP); anti-Cas9 (1:1,000, 7A9-3A3 14697, Cell Signalling); anti-FLAG (1:200, M2 clone, F1804, Sigma-Aldrich); anti-LAMIN B1 (1:500, ab16048, Abcam); anti-α-TUBULIN (1:3000, T6199, Sigma-Aldrich); anti-β-ACTIN (1:3,000, Abcam ab8227); and anti-VINCULIN (1:2,000, Abcam ab129002: Abcam); anti-histone H3 (1:7500, catalogue no. ab1791, Abcam); anti-LINE-1 ORF1p (1:1,000, clone 4H1, catalogue no. MABC1152, Merck); anti-LINE-1 ORF2p (1:1,000, MT49, a gift from Kathleen Burns), Anti-phospho-Histone H2A.X (Ser139) (1:1000, JBW301, 05-636, Merck).

Validation

Antibodies were validated by manufacturer or in previous publications as indicated below

anti-XPF D3G8C https://www.cellsignal.com/products/primary-antibodies/xpf-d3g8c-rabbit-mab/13465

anti-ERCC1 https://www.scbt.com/p/ercc1-antibody-d-10

anti-FANCA https://www.cellsignal.com/products/primary-antibodies/fanca-d1l2z-rabbit-mab/14657

anti-XPA https://www.cellsignal.com/products/primary-antibodies/xpa-d9u5u-rabbit-mab/14607

anti-FEN1 https://www.abcam.com/products/primary-antibodies/fen1-antibody-epr44602-ab109132.html

anti-SNM1A https://www.abcam.com/products/primary-antibodies/snm1a-antibody-ab226003.html

anti-SNM1B https://www.ptglab.com/products/DCLRE1B-Antibody-13203-1-AP.htm

anti-SLX1 PMID:19595721

anti-XRCC2 ptglab.com/products/XRCC2-Antibody-20285-1-AP.htm

anti-XRCC3 https://www.ptglab.com/products/XRCC3-Antibody-18494-1-AP.htm

anti-FLAG https://www.sigmaaldrich.com/GB/en/product/sigma/f1804

anti-Cas9 https://www.cellsignal.com/products/primary-antibodies/cas9-s-pyogenes-7a9-3a3-mouse-mab/14697

anti-β-ACTIN https://www.abcam.com/products/primary-antibodies/beta-actin-antibody-mabcam-8226-loading-control-ab8226.html

anti-VINCULIN https://www.abcam.com/products/primary-antibodies/vinculin-antibody-epr8185-ab129002.html

anti-histone H3 https://www.abcam.com/products/primary-antibodies/histone-h3-antibody-nuclear-marker-and-chip-grade-ab1791.html

anti-LINE-1 ORF1p clone 4H1 https://www.merckmillipore.com/GB/en/product/Anti-LINE-1-ORF1p-Antibody-clone-4H1,MM_NF-MABC1152

anti-LINE-1 ORF1p PMID:31892958

Anti-phospho-Histone H2A.X (Ser139): https://www.merckmillipore.com/GB/en/product/Anti-phospho-Histone-H2A.X-Ser139-Antibody-clone-JBW301,MM_NF-05-636

Eukaryotic cell lines

Policy information about cell lines and Sex and Gender in Research

Cell line source(s)

Joanna Wysocka provided K562 cells carrying the LINE-1 reporter. The Fanconi Anemia Research Foundation providing the PD20, PD220 and PD331 cell lines. SF-9 insect cells were obtained from Merck (89070101-1VL). All other lines were generated in this study.

Authentication	STR profiling
Mycoplasma contamination	All lines were mycoplasma free
Commonly misidentified lines (See ICLAC register)	No commonly misidentificatied cell lines were used in this study.

Animals and other research organisms

Policy information about studies involving animals; ARRIVE guidelines recommended for reporting animal research, and Sex and Gender in Research

Laboratory animals	All mice were maintained under specific pathogen-free conditions in individually ventilated cages (GM500; Techniplast) on Lignocel FS-14 spruce bedding (IPS) with environmental enrichment (fun tunnel, chew stick and Enviro-Dri nesting material (LBS)) at 19–23°C with light from 7:00 a.m. to 7:00 p.m., humidity 45-65%, and fed Dietex CRM pellets (Special Diet Services) ad libitum. No animals were wild and no field-collected samples were used. Mice were maintained on a C57BL/6J background. Embryos were used at E3.5 or E18.5 as indicated in the text. Samples were collected from animals at 8–12 weeks as specified in the text. Females used in timed mating experiments were aged between 6 and 18 weeks. The investigators were blinded to the genotypes of animals throughout the study and data were acquired by relying purely on identification numbers. Fancatm1a(EUCOMM)Wtsi (MGI ID: 4434431), Fancd2tm1Hou (MGI ID: 2673422), Ercc1tm1a(KOMP)Wtsi (MGI ID: 4362172), TnrTg(L1-EGFP)SN1Fhg (MGI ID: 244330), Tg(Zp3-cre)93Knw (MGI: 2176187) and Cgastm1d(EUCOMM)Hmgu (MGI ID: 2442261)
Wild animals	No wild animals were used
Reporting on sex	Reproductive tissues were examined therefore males and females were treated separately as detailed in the text
Field-collected samples	This study did not involve field-collected samples.
Ethics oversight	All animal experiments undertaken in this study were approved by the Medical Research Council's Laboratory of Molecular Biology animal welfare and ethical review body and the UK Home Office under the Animal (Scientific Procedures) Act 1986 (license no. PP6752216).

Note that full information on the approval of the study protocol must also be provided in the manuscript.

Flow Cytometry

Plots

Confirm that:

☒ The axis labels state the marker and fluorochrome used (e.g. CD4-FITC).

☒ The axis scales are clearly visible. Include numbers along axes only for bottom left plot of group (a 'group' is an analysis of identical markers).

☒ All plots are contour plots with outliers or pseudocolor plots.

☒ A numerical value for number of cells or percentage (with statistics) is provided.

Methodology

Sample preparation	The kidney, lung, femur and testes were isolated from adult mice and placed in cold PBS. To obtain single-cell suspensions from the kidney, the organ was chopped into small pieces in petri dishes and pipetted up and down with PBS. The tissue was recovered from the bottom of the tube and treated in 5mL HBSS containing 25mg collagenase II at 37 °C for 45 minutes. After that, the tissue was filtered through a 70 µm cell strainer, spun down and resuspended in FACS buffer (PBS supplemented with 2.5% FCS). For lung single-cell suspension, the procedure was identical but the pieces of tissue were treated with HBSS containing 25 mg collagenase II and 10 µg/mL DNAse for 1 hour. In the case of the bone marrow, cells were isolated from tibiae and femurs with the FACS buffer and strained through 70 µm cell strainers. Finally, testes were placed in a 100 mm culture dish containing 10 mL of PBS. Testicular tubules were separated from tunica albuginea and mechanically dissociated with forceps. Tubules were transferred to a 15 mL conical tube containing 5mL HBSS with 500 µL of 5 mg/mL collagenase IV and 25 µL of 10 mg/mL DNAse solution. This was incubated for 10 minutes at 37 °C. Then, the supernatant was discarded and tubules were collected and passed to a second conical tube containing 5mL HBSS with 500 µL of 5 mg/mL collagenase IV, 25 µL of 10 mg/mL DNAse solution and 25 µL of 10mg/mL hyaluronidase. This was incubated for 10 minutes at 37 °C, shaking the tube every 2 minutes to obtain a single-cell suspension. Cells were filtered in a 70 µm strainer, spun down and resuspended in the FACS buffer. Urogenital ridges of E12.5 embryos were isolated and placed into 150µl of trypsin solution (2.5µg/mL trypsin (Gibco), 25mM Tris, 120mM NaCl, 25mM KCl, 25mM KH2PO4, 25mM glucose, 25mM EDTA, pH 7.6) pre-warmed to 37°C and incubated for 10 minutes at 37°C. After that, 1µl of Benzonase endonuclease (Millipore) was added; disaggregation of the sample by gentle pipetting and incubated for a further 5minutes at 37°C. The trypsin was inactivated by adding 1ml of PBS/5% v/v fetal FCS. Following 10minutes of centrifugation at 3,300r.p.m., the cell pellet was resuspended in 100µl of Alexa Fluor 647-conjugated anti-human/mouse SSEA-1 antibody (catalogue no. MC-480; BioLegend) diluted 1:100 in staining buffer (PBS/2.5% v/v FCS) and incubated at room temperature for 10minutes; 300µl of staining buffer were added to the cell suspension

Instrument	LSRII analyzer (BD Biosciences)
Software	Data was collected using FACSDiva6.5 (BD) and processed using FlowJo v10.1r5.
Cell population abundance	N/A no samples were sorted
Gating strategy	For PGC quantification, cells were gated using FSC/SSC and PGCs defined as (APC-SSEA+GFP+)<br>For quantification of retrotranspositon events cells were gated using FSC/SSC and GFP + (R-670 v B-525)

☒ Tick this box to confirm that a figure exemplifying the gating strategy is provided in the Supplementary Information.
