# Supplementary figures and images for: Fanconi anemia DNA crosslink repair factors protect against LINE-1 retrotransposition during mouse development
Source: Nat Struct Mol Biol. 2023 Aug 14;30(10):1434–45. doi: 10.1038/s41594-023-01067-8 (PMC10584689; doi:10.1038/s41594-023-01067-8)

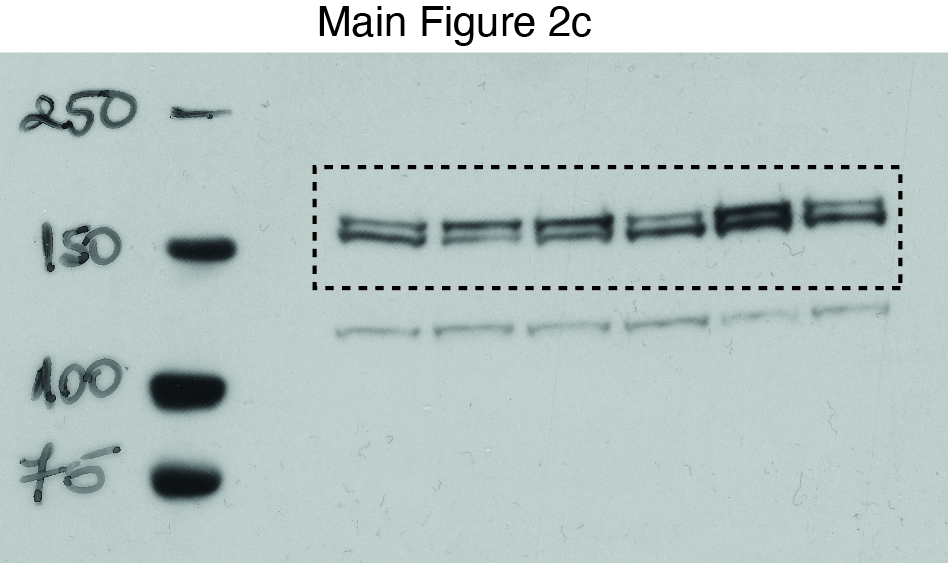

Supplement: Supplementary file 7 — Unprocessed western blots. [file 41594_2023_1067_MOESM7_ESM.jpg]

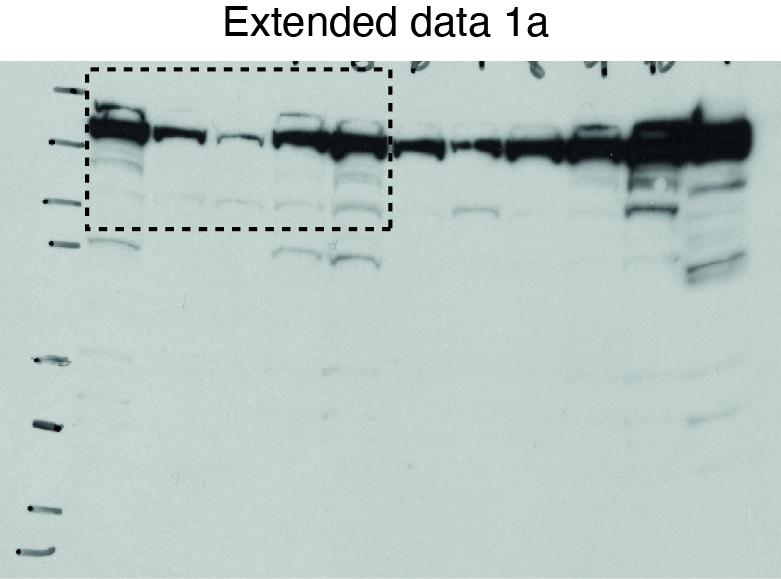

Supplement: Supplementary file 12 — Unprocessed western blots. [file 41594_2023_1067_MOESM12_ESM.jpg]

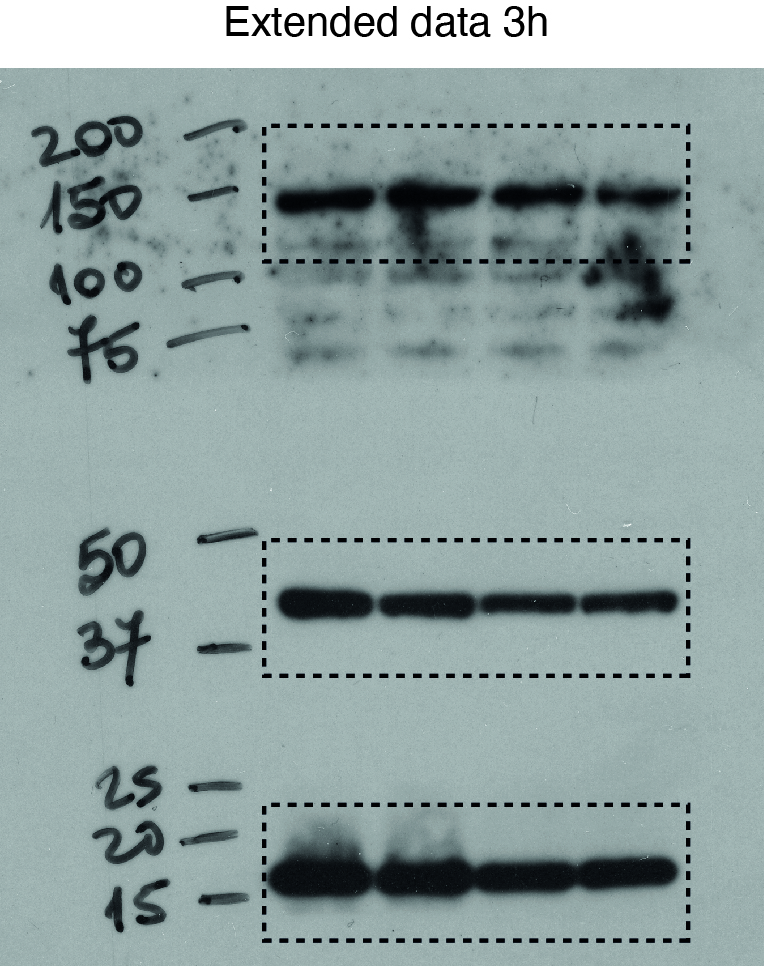

Supplement: Supplementary file 15 — Unprocessed western blots. [file 41594_2023_1067_MOESM15_ESM.jpg]

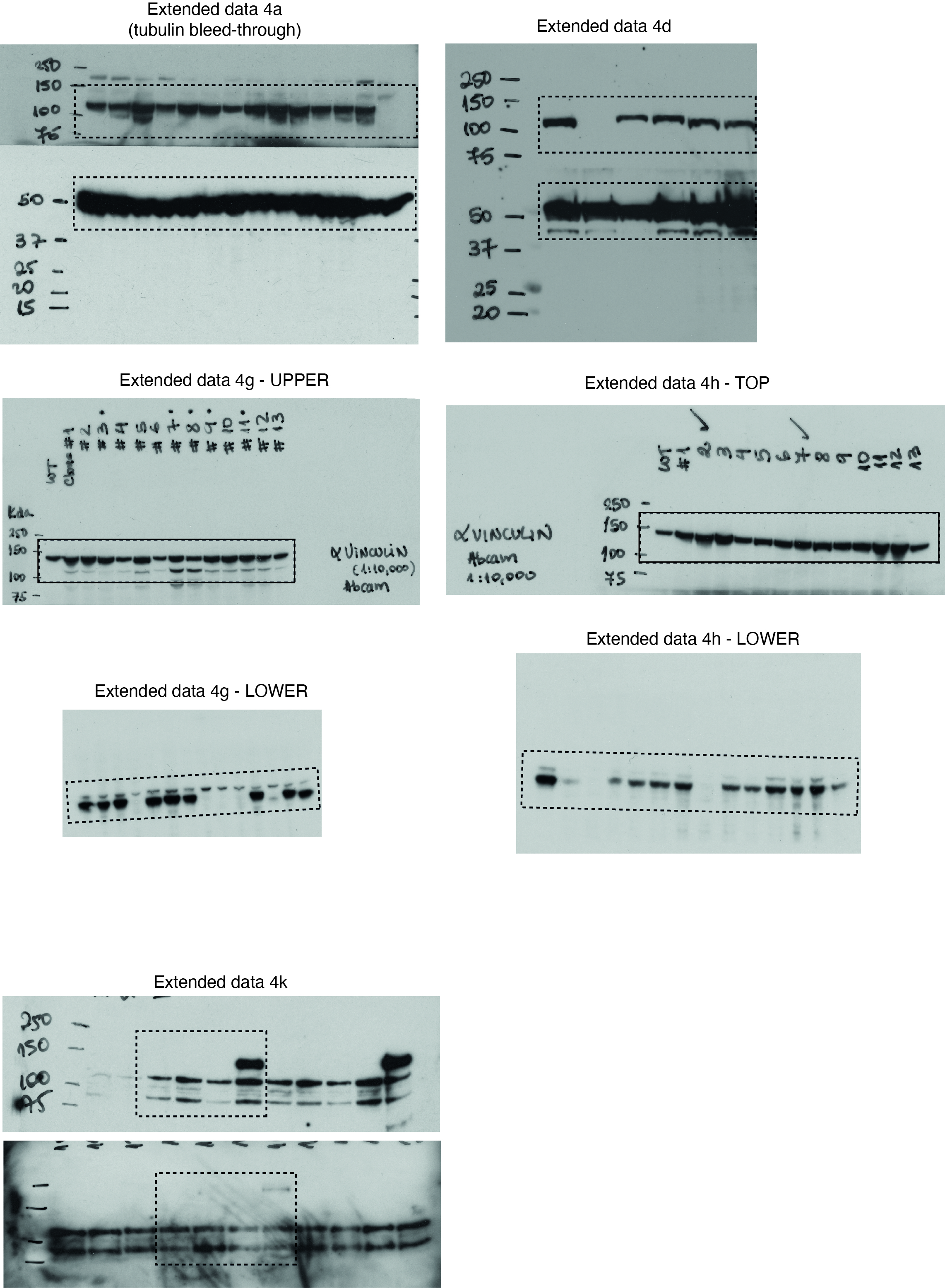

Supplement: Supplementary file 17 — Unprocessed western blots. [file 41594_2023_1067_MOESM17_ESM.jpg]
