# Supplementary material for: Fanconi anemia DNA crosslink repair factors protect against LINE-1 retrotransposition during mouse development
Source: Nat Struct Mol Biol. 2023 Aug 14;30(10):1434–45. doi: 10.1038/s41594-023-01067-8 (PMC10584689; doi:10.1038/s41594-023-01067-8)
Supplement: Supplementary file 1 — Supplementary Figs. 1–8. [file 41594_2023_1067_MOESM1_ESM.pdf]

nature structural & molecular biology

Article https://doi.org/10.1038/s41594-023-01067-8

Fanconi anemia DNA crosslink repair factors protect against LINE-1 retrotransposition during mouse development

In the format provided by the authors and unedited

Supplementary Figure 1 – Generation of Non-Homologous End Joining and BLM deficient K562 cells. Allele map showing sgRNA and the CRISPR–Cas9-mediated disruption sequences of the DNAPKcs locus for: **a)** sgRNA1 and **b)** sgRNA2. **c)** The cellular sensitivity of the DNAPKcs mutants generated by sgRNA1 and sgRNA2 to bleomycin (data represent mean and s.e.m.; *n*=3 independent experiments). Allele map showing sgRNA and the CRISPR–Cas9-mediated disruption sequences of the LIGASEIV locus for: **d)** sgRNA1 and **e)** sgRNA2. **f)** The cellular sensitivity of the LIGASEIV mutants generated by sgRNA1 and sgRNA2 to bleomycin (data represent mean and s.e.m.; *n*=3 independent experiments). Allele map showing sgRNA and the CRISPR–Cas9-mediated disruption sequences of the BLM locus for; **g)** sgRNA1 and **h)** sgRNA2. **i)** The cellular sensitivity of the BLM mutants generated by sgRNA1 and sgRNA2 to mitomycin C (data represent mean and s.e.m.; *n*=3 independent experiments).

Supplementary Figure 2 – Generation of Homologous Recombination mutants in K562 cells. Allele map showing sgRNA and the CRISPR–Cas9-mediated disruption sequences for: **a)** RAD51C, **b)** RAD54L, **c)** BRCA1 and **d)** BRCA2. **e)** The cellular sensitivity of the RAD51C and RAD54L mutants to bleomycin (data represent mean and s.e.m.; *n*=3 independent experiments). **f)** The cellular sensitivity of the BRCA1 and BRCA2 mutants to MMC (data represent mean and s.e.m.; *n*=3 independent experiments). **g)** Immunoblot in clones targeted XRCC2, VLC; vinculin loading control. **h)** Immunoblot in clones targeted XRCC3, VLC; vinculin loading control. **i)** The cellular sensitivity of the XRCC2 and XRCC3 mutants to MMC (data represent mean and s.e.m.; *n*=3 independent experiments). **j)** The cellular sensitivity of the XRCC2 and XRCC3 mutants to Olaparib (data represent mean and s.e.m.; *n*=3 independent experiments).

Supplementary Figure 3 – Generation of Translesion Synthesis and nuclease mutants in K562 cells. Allele map showing sgRNA and the CRISPR–Cas9-mediated disruption sequences for; **a)** REV1 and **b)** REV7. **c)** The cellular sensitivity of the REV1 and REV7 mutants to MMC (data represent mean and s.e.m.; *n*=3 independent experiments). Immunoblot in clones targeted for; **d)** FEN1, **e)** SLX1, **f)** SNM1B, **g)** SNM1A; VLC, vinculin loading control; BACT, beta-actin loading control (representative data from 2 independent experiments). Allele map showing sgRNA and the CRISPR–Cas9-mediated disruption sequences of the XPF locus for; **h)** sgRNA1 and **i)** sgRNA2. Allele map showing sgRNA and the CRISPR–Cas9-mediated disruption sequences for: **j)** MUS81 and **k)** FAN1. **l)** Immunoblot in clones in which targeted XPF in various genetic backgrounds; TUB, *α*-tubulin loading control; EN-, K562 L1(D205A)-G418^R (endonuclease dead) (representative data from 2 independent experiments). **m)** The cellular sensitivity of the K562 L1-G418^R (endonuclease proficient) XPF mutants generated with sgRNA1 and sgRNA2 to mitomycin C (data represent mean and s.e.m.; *n*=3 independent experiments). **n)** The cellular sensitivity of the MUS81 and FAN1 mutants to mitomycin C (data represent mean and s.e.m.; *n*=3 independent experiments).

Supplementary Figure 4 – Generation of FA ICL repair and XPA deficient K562 cells. a) Immunoblot in clones targeted for FANCA with sgRNA1 or sgRNA2 in wildtype or mutant K562 L1-G418^R and K562 L1(D205A)-G418^R cells; TUB, *α*-tubulin loading control (representative data from 2 independent experiments). **b)** The cellular sensitivity of the K562 L1-G418^R FANCA mutants generated by sgRNA1 and sgRNA2 to mitomycin C (data represent mean and s.e.m.; *n*=3 independent experiments). **c)** Immunoblot in clones targeted for XPA with sgRNA1 or sgRNA2 in wildtype K562 L1-G418^R cells; TUB, *α*-tubulin loading control (representative data from 2 independent experiments). **d)** The cellular sensitivity of the XPA mutants generated with sgRNA1 and sgRNA2 to UV irradiation (data represent mean and s.e.m.; *n*=3 independent experiments). **e)** Immunoblot in clones targeted for FANCD2 with sgRNA1 or sgRNA2 in wildtype or mutant K562 L1-G418^R and K562 L1(D205A)-G418^R cells; FANCI loading control (representative data from 2 independent experiments). **f)** The cellular sensitivity of the K562 L1-G418^R FANCD2 mutants generated with sgRNA1 and sgRNA2 to mitomycin C (data represent mean and s.e.m.; *n*=3 independent experiments). **g)** Allele map showing sgRNA1 and sgRNA2 and the CRISPR–Cas9-mediated disruption sequences for SLX4 allele. **h)** The cellular

sensitivity of the SLX4 mutants to mitomycin C (data represent mean and s.e.m.; *n*=3 independent experiments). Immunoblot for FANCD2 in *ΔFANCD2* (sgRNA1) clones ectopically expressing either **i)** YFP-FANCD2 or **j)** YFP-FANCD2(K516R) mutant cDNA. Vinculin loading control (representative data from 2 independent experiments).

Supplementary Figure 5 – Generation of DNA repair deficient mutants in K562 cells carrying endonuclease deficient L1-G418 ORF2-D205A reporter. Allele map showing sgRNA and the CRISPR–Cas9-mediated disruption sequences for; **a)** REV1 and **b)** REV7 in the K562 L1(D205A)-G418^R cell line. **c)** The cellular sensitivity of the REV1 and REV7 mutants in the K562 L1(D205A)-G418^R cell line to methyl-methanesulfonate (MMS) (data represent mean and s.e.m.; *n*=3 independent experiments). Allele map showing sgRNA and the CRISPR–Cas9-mediated disruption sequences for: **d)** BRCA1 and **e)** BRCA2 in the endonuclease dead L1-G418 ORF2-D205A (EN-) K562 reporter cell line. **f)** The cellular sensitivity of the BRCA1 and BRCA2 mutants in the K562 L1(D205A)-G418^R cell line to mitomycin C (data represent mean and s.e.m.; *n*=3 independent experiments). Allele map showing sgRNA and the CRISPR–Cas9-mediated disruption sequences for; **g)** MUS81, **h)** FAN1 and **i)** SLX4 in the endonuclease dead K562 L1(D205A)-G418^R cell line. **j)** The cellular sensitivity of the SLX4, FAN1 and MUS81 mutants in the endonuclease dead K562 L1(D205A)-G418^R cell line to mitomycin C (data represent mean and s.e.m.; *n*=3 independent experiments).

Supplementary Figure 6 – SN1 LINE-1 reporter and DNA repair factors expression in adult mice. **a)** Schema illustrating the strategy to measure the expression of the SN1 LINE-1 reporter. A Taqman probe was used to target the ORF2p region of the reporter. **b)** The expression of SN1 LINE-1 reporter *(Sn1 Line-1 Orf2p)* was quantified by RT-qPCR in multiple tissues from adult mice. Expression was normalized to *Gapdh* and made relative to the heart sample. Data represents the mean, error bars represent the s.e.m., *n*=5 mice. **c)** The expression of different DNA repair factors (*Fanca, Slx4 and Ercc1*) was quantified by RT-qPCR in multiple tissues from adult mice. Expression was normalized to *Gapdh* and made relative to the intestine sample. Data represents the mean, error bars represent the s.e.m., *n*=3 mice. **d)** The expression of SN1 LINE-1 reporter *(Sn1 Line-1 Orf2p)* was quantified by RT-qPCR in multiple tissues from *SN1*, *SN1 Ercc1*-deficient and *SN1 Fanca*-deficient adult mice. Expression was normalized to *Gapdh* and made relative to *SN1* in each organ. Data represents the mean, error bars represent the s.e.m., *n*=3 mice.

Supplementary Figure 7 – Flow cytometry gating strategies. Gating strategies for quantification of GFP^+ cells in single-cells derived from: **a)** kidney, **b)** bone marrow, **c)** lung, **d)** testis, **e)** PGCs.

Supplementary Figure 8 – Uncropped western blots.

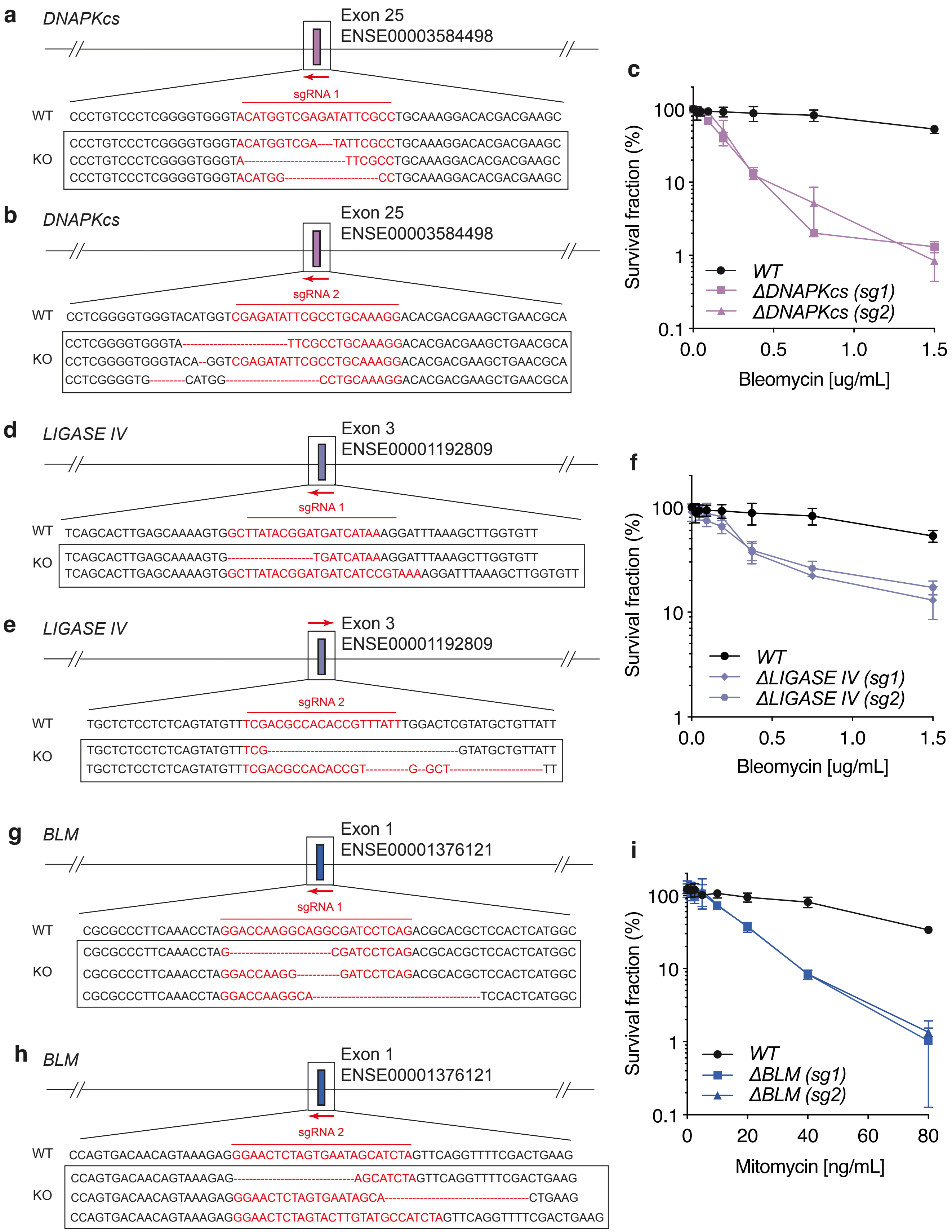

Supplementary Figure 1 – Generation of Non-Homologous End Joining and BLM deficient K562 cells

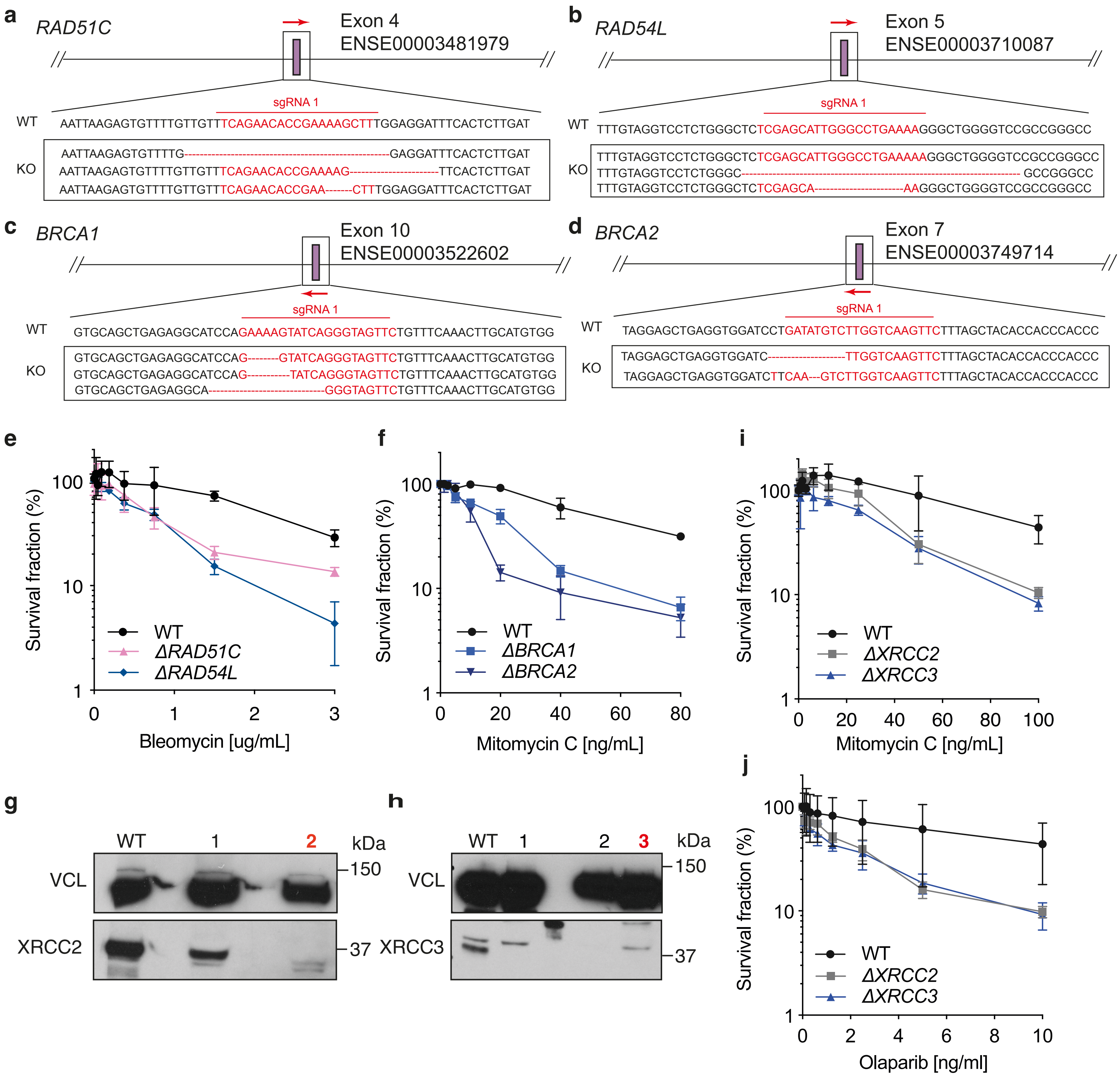

Supplementary Figure 2 – Generation of Homologous Recombination mutants in K562 cells

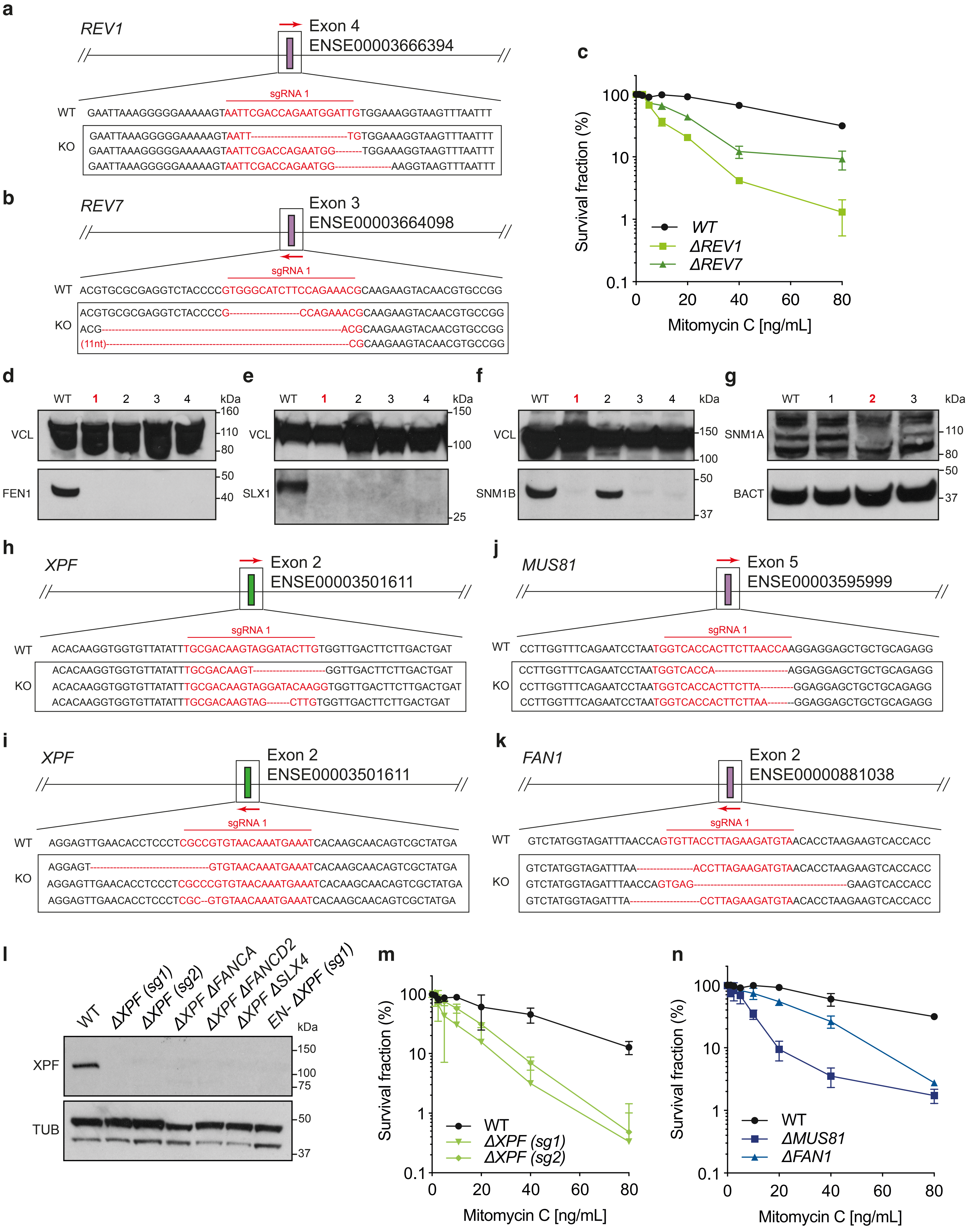

Supplementary Figure 3 – Generation of translesion synthesis and nuclease mutants in K562 cells

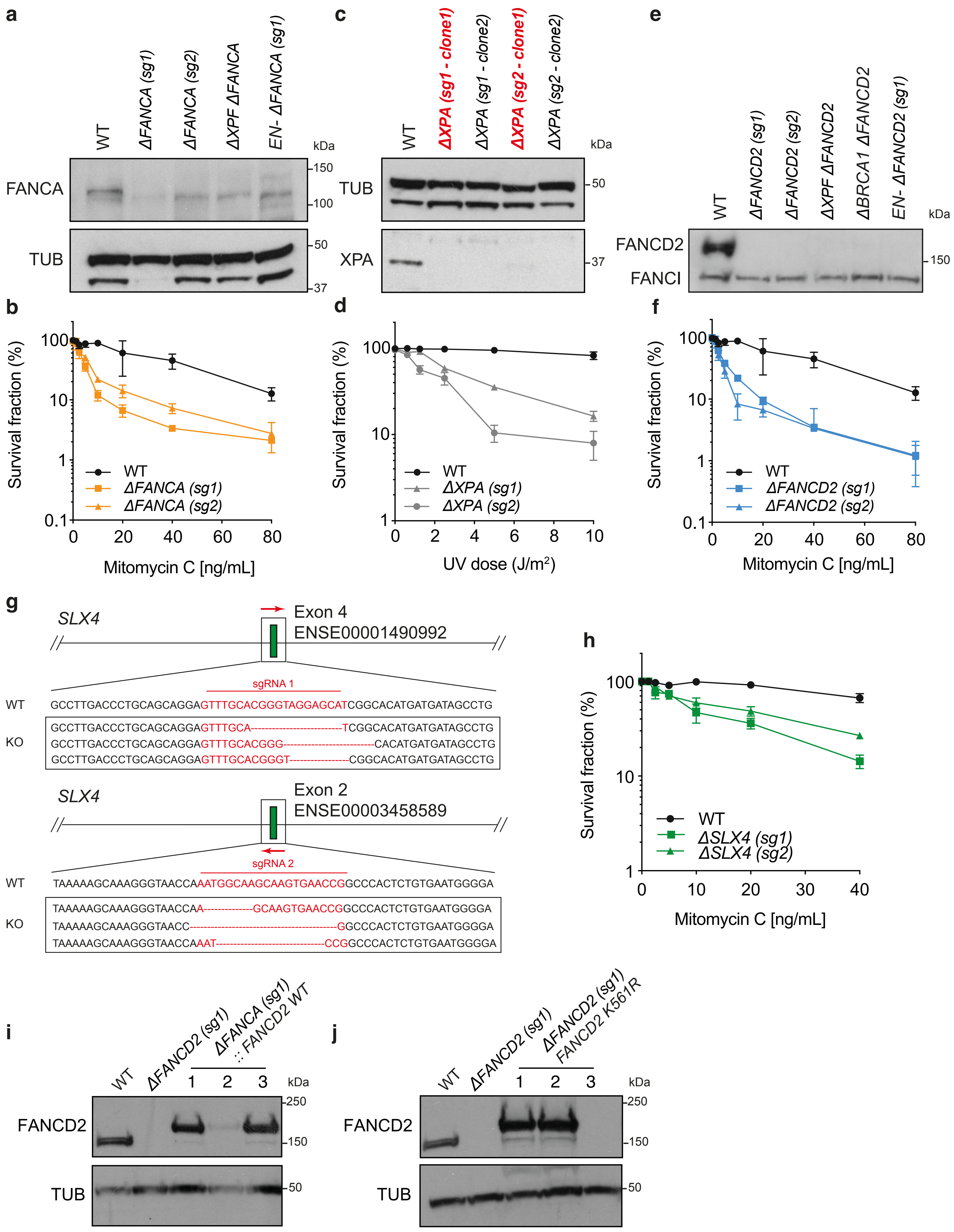

Supplementary Figure 4 – Generation of FA ICL repair and XPA deficient K562 cells

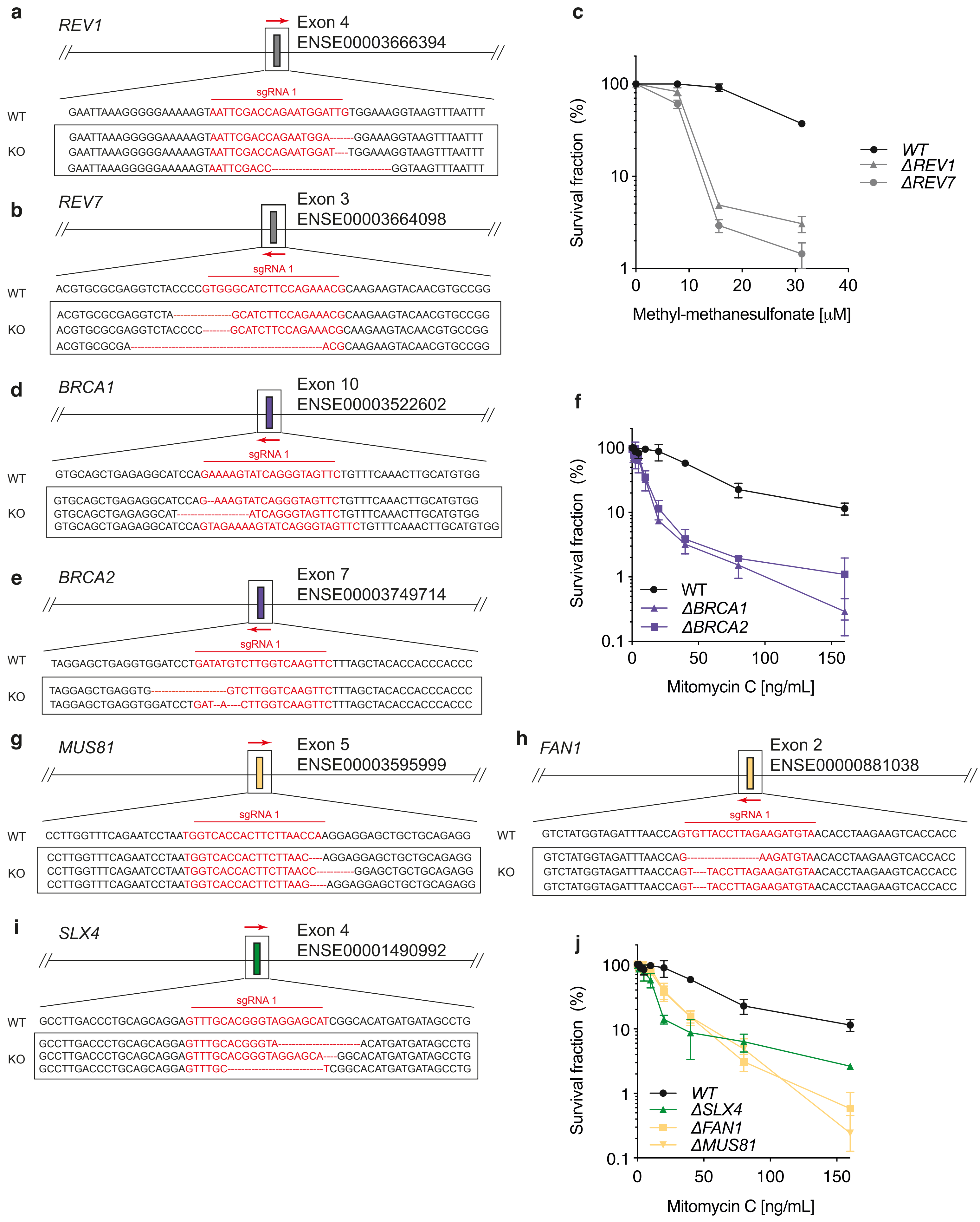

Supplementary Figure 5 – Generation of DNA repair deficient mutants in K562 cells carrying endonuclease deficient L1(D205A)-G418 reporter

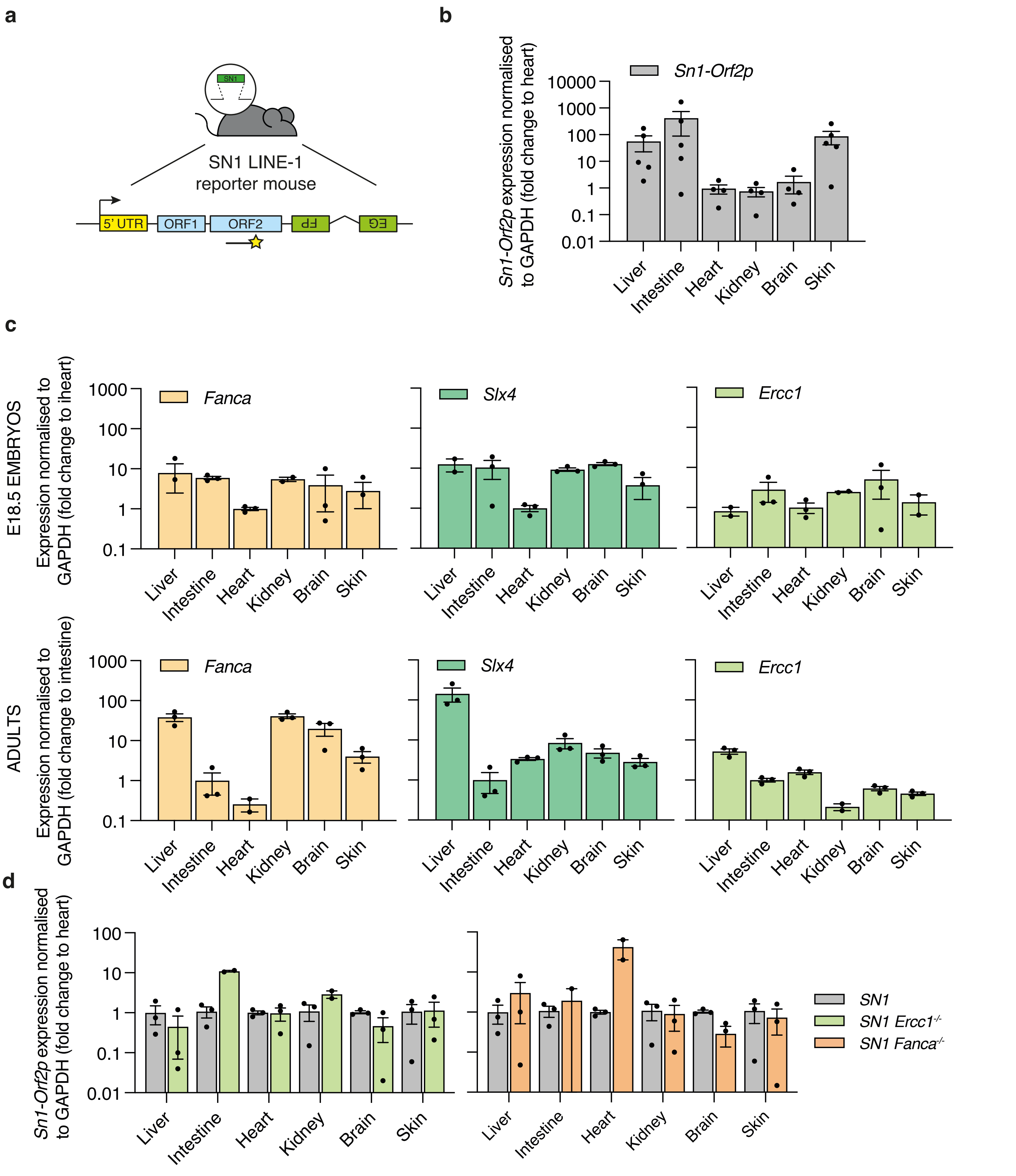

Supplementary Figure 6 – SN1 LINE-1 reporter and DNA repair factors expression in mice

a

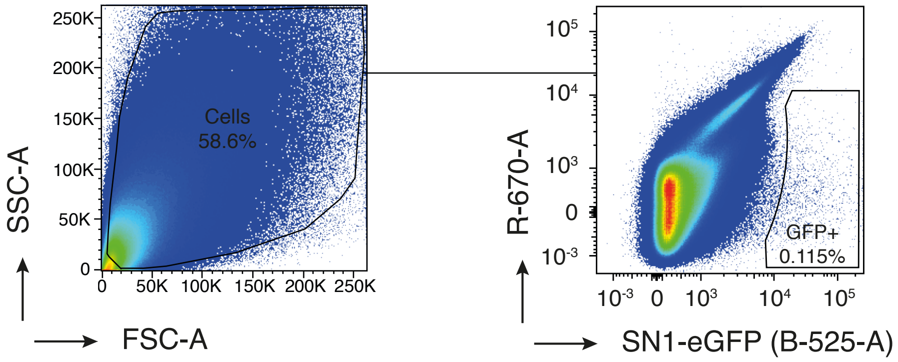

Gating strategy for quantification of GFP+ cells in kidney, accompaying Figures 4f, g and Supplementary Figure 14a, b

b

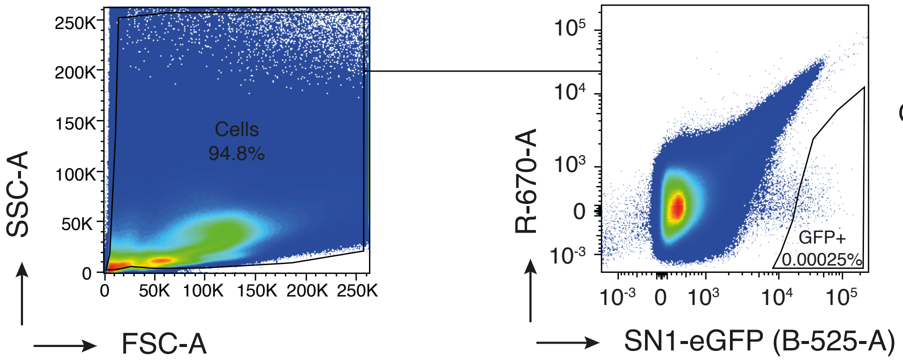

Gating strategy for quantification of GFP+ cells in bone marrow, accompaying Supplementary Figure 14c, d, i

c

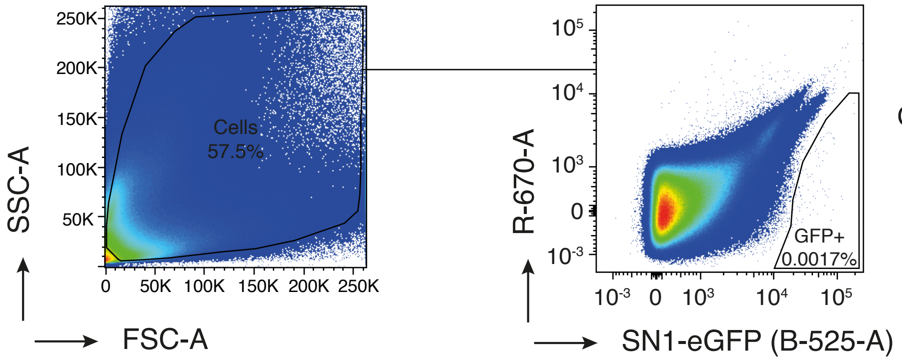

Gating strategy for quantification of GFP+ cells in lung, accompaying Supplementary Figure 14e, f

d

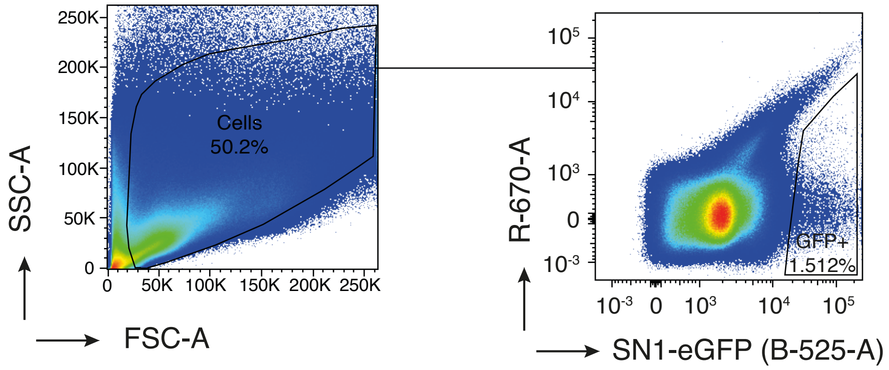

Gating strategy for quantification of GFP+ cells in testis, accompaying Figures 4h, i and Supplementary Figure 14g, h, i

e Gating strategy for quantification of PGCs, accompaying Supplementary Figure 11i, j

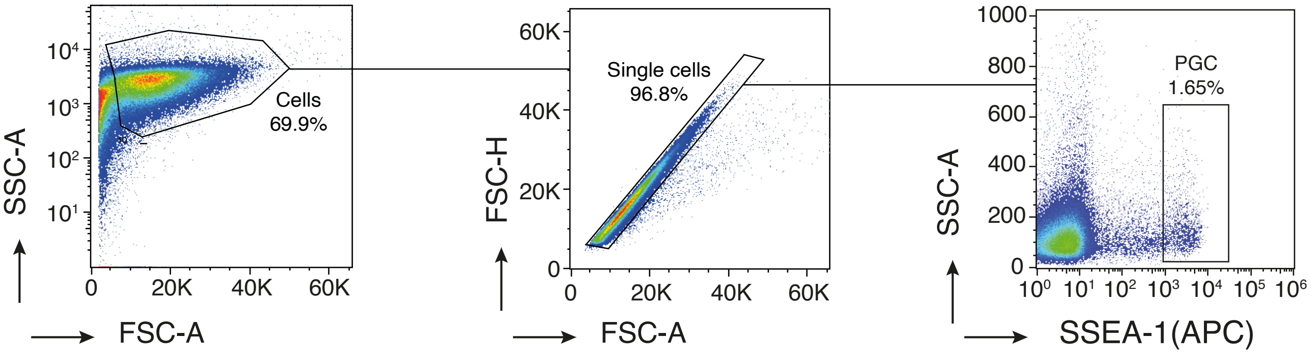

Supplementary Figure 7 – Flow cytometry gating strategies

Supplementary Figure 2i

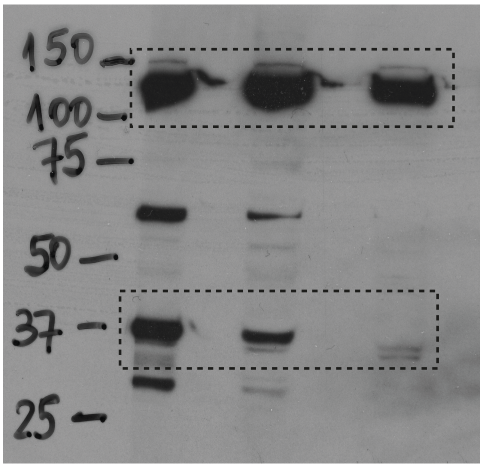

Supplementary Figure 2j

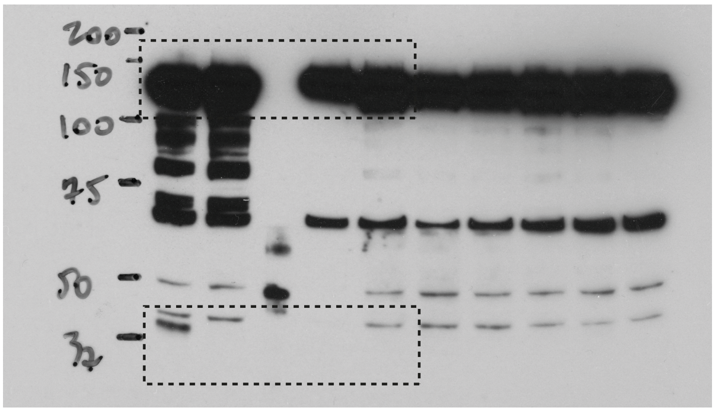

Supplementary Figure 3d

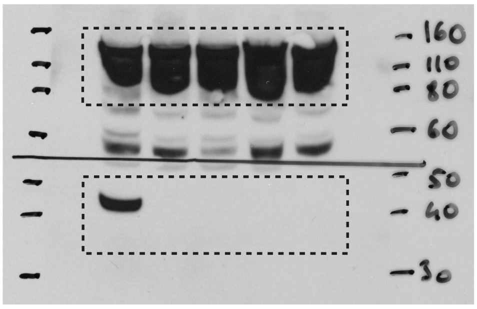

Supplementary Figure 3e

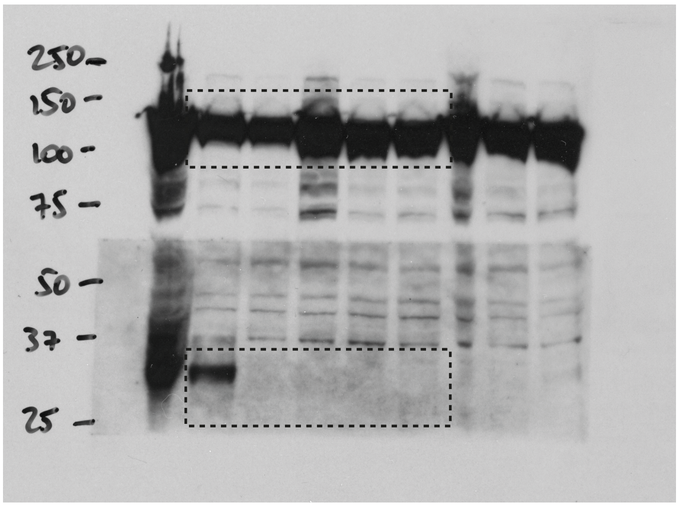

Supplementary Figure 3f

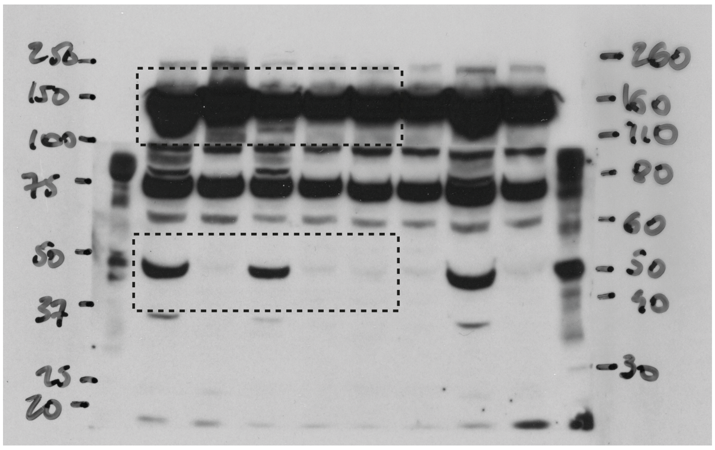

Supplementary Figure 3g

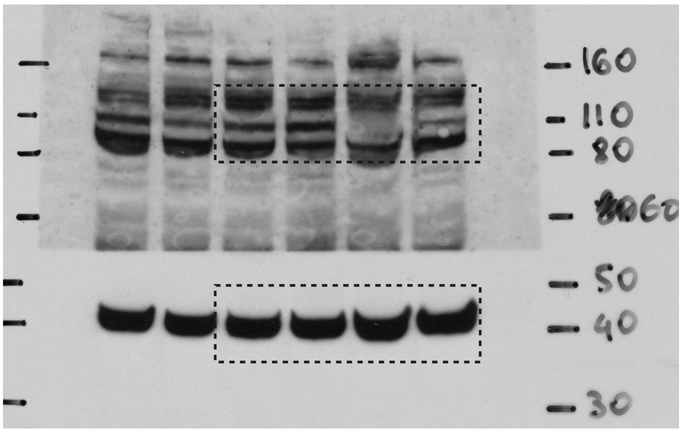

Supplementary Figure 8 – Uncropped western blots

Supplementary Figure 3l (tubulin bleed-through)

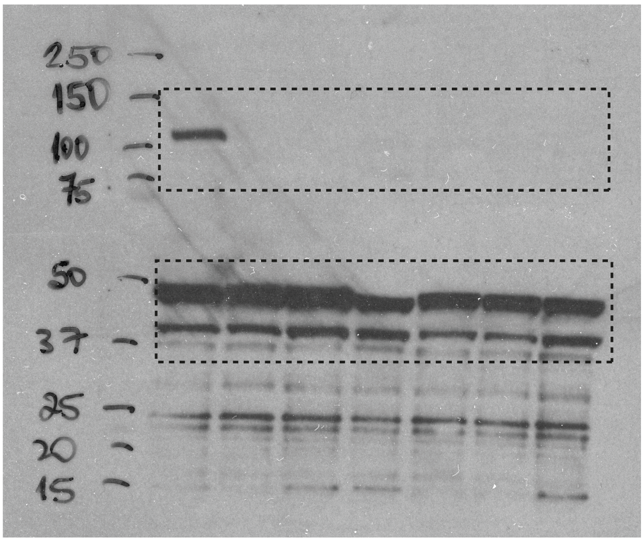

Supplementary Figure 4b
(tubulin bleed-through)

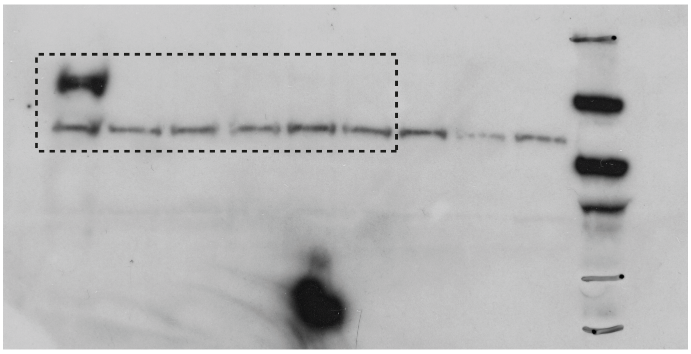

Supplementary Figure 4a
(tubulin bleed-through)

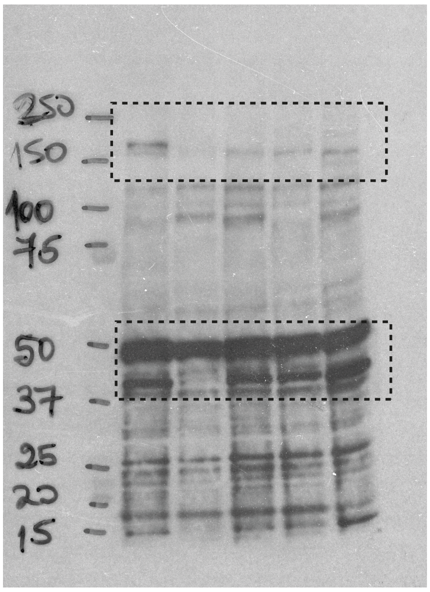

Supplementary Figure 4c
(tubulin bleed-through)

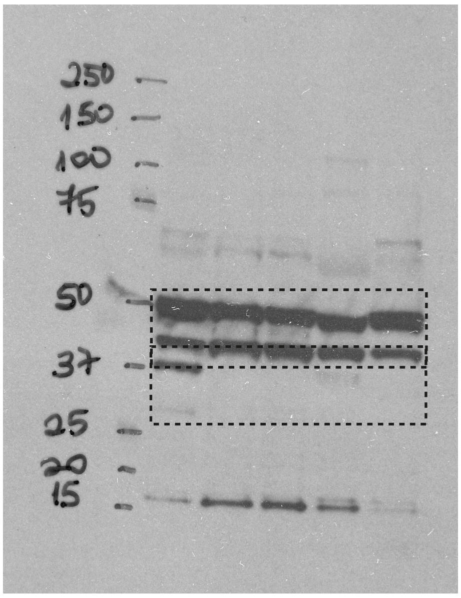

Supplementary Figure 4i

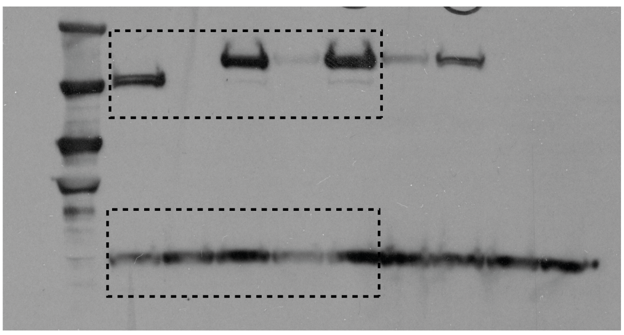

Supplementary Figure 4j

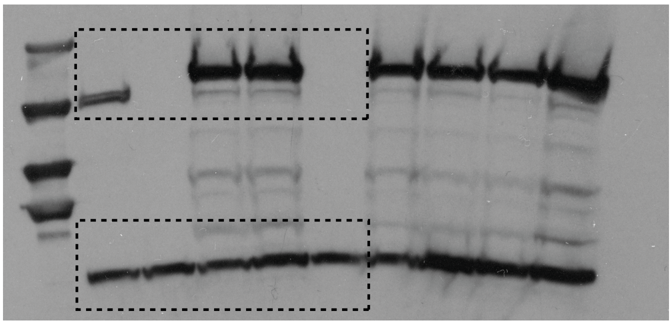

Supplementary Figure 8 – Uncropped western blots
